# STING-Licensed Macrophages Prime Type I IFN Production by Plasmacytoid Dendritic Cells in the Bone Marrow during Severe *Plasmodium yoelii* Malaria

**DOI:** 10.1371/journal.ppat.1005975

**Published:** 2016-10-28

**Authors:** Emily Spaulding, David Fooksman, Jamie M. Moore, Alex Saidi, Catherine M. Feintuch, Boris Reizis, Laurent Chorro, Johanna Daily, Grégoire Lauvau

**Affiliations:** 1 Albert Einstein College of Medicine, Department of Microbiology and Immunology, Bronx, NY, United States Of America; 2 Albert Einstein College of Medicine, Department of Pathology, Bronx, NY, United States Of America; 3 University of Malawi College of Medicine, Blantyre Malaria Project, Blantyre, Malawi; 4 Albert Einstein College of Medicine, Department of Medicine, Division of Infectious Diseases, Bronx, NY, United States Of America; 5 New York University Medical Center, Department of Pathology and Department of Medicine, New York, NY, United States Of America; University of Medicine and Dentistry of New Jersey, UNITED STATES

## Abstract

Malaria remains a global health burden causing significant morbidity, yet the mechanisms underlying disease outcomes and protection are poorly understood. Herein, we analyzed the peripheral blood of a unique cohort of Malawian children with severe malaria, and performed a comprehensive overview of blood leukocytes and inflammatory mediators present in these patients. We reveal robust immune cell activation, notably of CD14^+^ inflammatory monocytes, NK cells and plasmacytoid dendritic cells (pDCs) that is associated with very high inflammation. Using the *Plasmodium yoelii 17X YM* surrogate mouse model of lethal malaria, we report a comparable pattern of immune cell activation and inflammation and found that type I IFN represents a key checkpoint for disease outcomes. Compared to wild type mice, mice lacking the type I interferon (IFN) receptor exhibited a significant decrease in immune cell activation and inflammatory response, ultimately surviving the infection. We demonstrate that pDCs were the major producers of systemic type I IFN in the bone marrow and the blood of infected mice, via TLR7/MyD88-mediated recognition of *Plasmodium* parasites. This robust type I IFN production required priming of pDCs by CD169^+^ macrophages undergoing activation upon STING-mediated sensing of parasites in the bone marrow. pDCs and macrophages displayed prolonged interactions in this compartment in infected mice as visualized by intravital microscopy. Altogether our findings describe a novel mechanism of pDC activation *in vivo* and precise stepwise cell/cell interactions taking place during severe malaria that contribute to immune cell activation and inflammation, and subsequent disease outcomes.

## Author Summary

The *Plasmodium* parasite is the number one killer among human parasitic diseases worldwide. Protection is associated with length of exposure for people living in endemic areas, with severe disease primarily affecting young children. Inflammation is a key component in the pathophysiology in malaria, and disease severity has been linked to the degree of activation of the immune system. However, the underlying mechanisms of protection and disease outcomes remain poorly understood. We provide a comprehensive analysis of peripheral blood immune cells obtained from a cohort of children with severe malaria. Our results show heightened inflammation and immune cell activation, in particular for monocytes, natural killer cells, and plasmacytoid dendritic cells (pDCs). We have also utilized a mouse model of lethal malaria that recapitulates many features identified in this cohort of severe malaria patients to examine drivers of immune cell activation and inflammation. Our studies provide evidence that type I interferon (IFN) acts as an early switch in inducing a potent inflammatory response in the infected host. Type I IFN production is massively produced in the bone marrow and the blood of infected mice by plasmacytoid dendritic cells (pDCs), a subset of DCs. We also demonstrate that resident macrophages in the bone marrow, control type I IFN production by the pDCs. We define how both myeloid cells “sense” the parasite to initiate the host immune response and report a previously uncharacterized physical interaction between pDCs and macrophages in the bone marrow as visualized by intravital microscopy *in vivo*. Our results define cellular processes underlying the marked inflammation of severe malaria and could open novel therapeutic opportunities to improve outcomes in this important human infectious disease.

## Introduction

Malaria still remains a significant global health problem worldwide with 214 million people infected (range 149–303 million, WHO 2015), and half a million deaths each year [1, 2]. Death occurs as a result of severe malaria, a multi-systemic disorder resulting from blood stage *Plasmodium* infection that is associated with significant morbidity and high case fatality rates despite antimalarial treatment [1, 3]. It primarily affects young children in endemic areas and travelers, both lacking exposure-induced immunity. Severe malaria includes cerebral malaria (CM), severe anemia and metabolic acidosis and is characterized by extensive parasitized erythrocyte sequestration in CM and subsequent organ dysfunction with a heightened and dysregulated immune response that remains poorly understood [4]. Lack of a highly effective vaccines and emergence of artemisinin resistance limits malaria control measures. Thus additional efforts to understand severe disease with the goal of improving clinical outcomes remains a very high priority [5, 6].

Severe malaria patients exhibit high levels of serum pro-inflammatory cytokines (TNF, IL-1β, IL-6, IL-8, IL-12, IFNγ) and chemokines (CCL2, CCL5, CXCL9, CXCL10), and lower levels of regulatory cytokines (IL-10, TGFβ, PGE2) [7] compared to those with mild and asymptomatic malaria [8–13]. Yet the mechanisms driving the high inflammatory states in severe malaria are not well understood. The type I interferon (IFN) cytokine is particularly interesting as it is released very rapidly upon sensing of microbial products and exhibits broad immunomodulatory properties [14, 15]. Upregulation of the type I IFN pathway has been associated with mild malaria patients compared to severe disease patients [16] and polymorphisms in the type I IFN receptor with severe disease outcomes [17, 18]. Age-associated resistance against severe malaria infection–both for *P*. *falciparum* and *P*. *vivax*- and asymptomatic infection, directly correlates with high levels of regulatory cytokines [7, 10]. In this context, a role for Foxp3^+^ CD4^+^ regulatory T cells (Tregs) as well as non-Foxp3^+^ activated/memory (CD45RO^+^) CD4^+^ T cells in regulating malaria-induced inflammation and subsequent immunopathology is supported by many studies in human patients and in surrogate mouse models [19–24]. NK and γδ T cell activation is increased in children suffering from CM compared to other forms of malaria, as is the proportion of activated/memory T cells [25]. Inflammatory CCR2^+^ CD14^+^ monocytes mediate antibody-dependent cellular inhibition, and their higher proportion was correlated to lower blood parasitemia in acute uncomplicated malaria patients [9]. While most cited studies have focused on one or another immune cell type, there is only relatively limited information that exists on a global analysis of the leukocyte response taking place in the blood of patients undergoing severe malaria.

Initiation of a systemic immune response that follows the sensing of blood *Plasmodium* parasites is a poorly understood process that involves multiple pathways triggered by parasite-derived molecules and byproducts, and a subsequent rapid inflammatory response. Major *Plasmodium* parasite molecules sensed by the host immune system include glycophosphatidylinositol (GPI), haemozoin crystals and nucleic acids. GPI anchors trigger TLR2/TLR1 and TLR2/TLR6 heterodimers -and to some extent TLR4-, leading to the production of pro-inflammatory cytokines (TNF, IL-1β) via MyD88 [26, 27], reactive nitrogen and oxygen species [28, 29], and the upregulation of adhesion molecules (ICAM1, VCAM1)[30]. Haemozoin crystals result from haemoglobin digestion by blood stage *Plasmodium* parasites which stimulate massive secretion of IL-1β, TNF and chemokines. Haemozoin alone [31, 32] or complexed to parasite DNA [33] triggers TLR9, ultimately leading to phago-lysosomal destabilization and cytosolic sensing of parasite DNA via AIM2 and haemozoin via NLRP3 inflammasomes inside phagocytes [34]. The STING pathway has also been implicated in the recognition of AT-rich stem loop motifs of *Plasmodium* DNA inside the cytosol of macrophages *in vitro*, driving TBK1/IRF3-mediated secretion of type I IFN [35]. These studies were primarily conducted on macrophages cell lines *in vitro*, on *ex vivo* isolated or *in vitro* grown myeloid cells, and/or made use of parasite-derived or synthetic ligands. Other reports analyzed the role of these pathways using various surrogate mouse models of malaria and mice lacking the tested pathways [35–37]. Most commonly used models in these studies included non-lethal acute *P*. *yoelii (Py)* (17XNL) or chronic *P*. *chabaudi (Pc)*, as well as lethal *Py* (17X YM) and *P berghei* (ANKA) respectively as models of high parasitemia and of CM. Along with the human reports, results from these investigations suggested that these sensing receptors and pathways promote high inflammatory response to the parasite and often poor outcomes. For instance, mice lacking MyD88, the common adaptor protein for many *Plasmodium*-stimulated TLRs, exhibited diminished blood inflammatory cytokines (IL-6, IL-12, IFNα, IFNγ, TNF, CCL2) in several of the murine models, leading to improved resistance to these infections [38, 39]. Interestingly, single TLR deficiencies, such as in TLR2, TLR4, TLR9 or TLR7, only led to mild differences compared to WT mice, suggesting that multiple sensing mechanisms converge in mediating host resistance to the parasite. Similarly to MyD88^-/-^ mice, mice lacking a functional cytosolic adaptor protein STING, which controls multiple cytosolic sensing receptors driving the induction of type I IFN, exhibited increased resistance to *P*. *berghei* infection [35]. Consistent with the potential importance of type I IFN, lack of type I IFN production in mice deficient for the type I IFN transcriptional regulators IRF3 and IRF7 [35], or lack of type I IFN signaling in mice disrupted for the IFNα/β receptor, enabled the survival of *P*. *berghei* infected mice [17, 37]. Production of type I IFN was also suggested to involve TLR9/ and TLR7/MyD88, and IRF7 in splenic macrophages and pDCs, promoting enhanced early immune cell activation and pro-inflammatory response (IL-12, IFNγ, TNF) in *P*. *chabaudi* infection [36, 40, 41]. Yet none of the mice lacking these sensors or type I IFN signaling exhibited substantial clinical phenotypes in non-lethal models, consistent with the idea that clinical outcomes are regulated by the nature of *Plasmodium* infection, e.g., mouse genetic background and strain of *Plasmodium*.

Classical DCs and pDCs [42], macrophages [36, 43] and CCR2^+^ monocytes [44] are important contributors to *Plasmodium*-induced inflammation, through secretion of type I IFN and other inflammatory mediators (IL-12, TNF, IL-6) and chemokines, production of antimicrobial effector proteins (reactive oxygen and nitrogen species) and activation of robust T cell responses [45]. For instance, early type I IFN signaling in DCs activated following *P*. *berghei* infection, suppressed their ability to prime strong protective "Th1" CD4^+^ T cell responses [37, 46], preventing the control of infection and host survival.

While above referenced studies have clearly highlighted key innate sensing pathways and cytokines, and the implication of specific subsets of cells in detecting *Plasmodium* parasites *in vivo*, the precise initiating steps which include the tissue, the cell subsets and the cell-intrinsic sensing mechanisms and their relative contribution remains to be further defined. The spleen for instance, represents an essential organ in the course of blood stage malaria infections, both in humans and mice, largely because of its central function in inducing immune responses, monitoring blood through the removal of damaged RBCs and iron recycling, and its ability to produce emergency extramedullary haematopoeisis [47]. Splenectomized patients, similarly to that of non-lethal murine malaria, exhibit higher parasitemia, yet in lethal *P*. *yoelii* host resistance is improved, suggesting a deleterious contribution of spleen-derived inflammation. Whether other organs and cells play a key role during blood stage malaria has not yet been thoroughly addressed. The systemic nature of this infection would support the hypothesis that many organs and myeloid cells residing in these tissues are likely to contribute to shape infection outcomes. Recent reports analyzing changes in the bone marrow hematopoiesis during *P*. *chabaudi* infection illustrated the existence of major modifications occurring in this compartment, with a temporary loss of myeloid-erythroid precursors and the common lymphoid progenitor, and the emergence of a new, infection-induced population of atypical progenitor cells (IL-7Rα^+^ c-Kit^hi^) with lymphoid and myeloid potential [48]. Whether the bone marrow plays additional roles in initiating the immune response against the parasite requires further investigations.

In summary, multiple cellular sources for key inflammatory factors such as type I IFN have been proposed, and these notably include macrophages, DCs and pDCs [36, 41, 49]. However, little is known as far as (i) which immune cells first sense infected iRBCs and/or parasite-derived immunogens and (ii) which cell-intrinsic molecular pathways are involved. The precise cell/cell interactions taking place during active blood stage malaria infection *in vivo*, the relative contributions of the various cell subsets to these key processes, and the sequences of events that occur and in which tissues have not been determined. Such information is acutely lacking and is absolutely essential to gain understanding of this important global infectious disease, and to possibly design novel therapeutic strategies.

In the current study we have made use of lethal *P*. *yoelii 17X YM* as surrogate model of severe malaria to investigate these relevant mechanistic questions. Two major murine models of severe malaria, *P*. *yoelii* and *P*. *berghei*, recapitulating some of the key immunological features reported in patients experiencing severe malaria, are available, yet substantially less information is available on *Py*-infected mice. We first conducted an extensive phenotypic and functional analysis of immune cells and inflammatory factors on all peripheral blood leukocytes isolated from a unique cohort of Malawian children undergoing severe malaria. A parallel analysis conducted on the blood of *Py-*infected mice, largely reflected the high inflammatory state and immune cell activation observed in the human patients. We show this broad activation of leukocytes, in particular Ly6C^hi^ monocytes, NK cells and pDCs is accounted for by an early systemic burst of type I IFN. Type I IFN is acting as a key initiating cytokine orchestrating the robust inflammatory syndrome and the lethal outcomes. We define (i) pDC as the major producers of systemic type I IFN in the bone marrow and the blood, (ii) pDC activation requirements by macrophages in the bone marrow and their physical interactions, and (iii) the *Plasmodium* parasite sensing mechanisms that occur via TLR7/MyD88 in pDCs and STING in macrophages.

## Results

### Parallel systemic inflammation and innate immune cell activation in the blood of patients and mice with severe malaria

**Fig 1:**
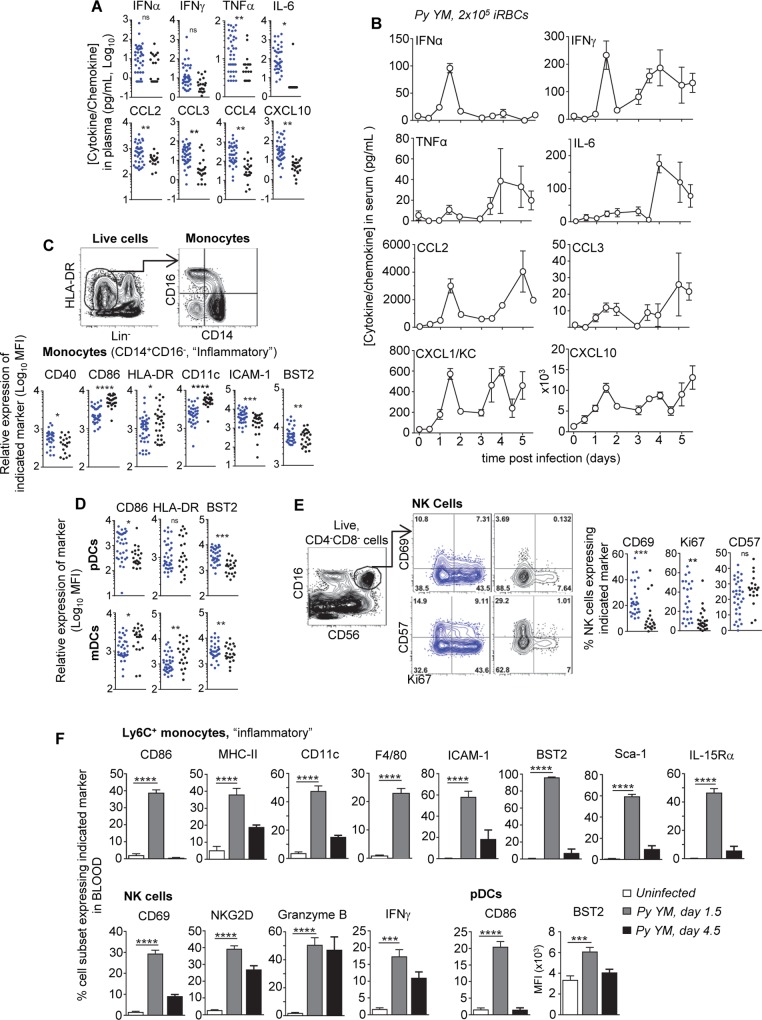
High inflammation and immune cell activation in the blood of severe malaria patients and in a surrogate mouse model. (**A**) Serum cytokine and chemokine levels in patients. Each point represents an individual patient, enrollment (blue), follow-up (black). (**B**) WT B6 mice were inoculated i.v. with 2x10^5^ *Py 17X YM* iRBCs. Kinetics of cytokine and chemokine levels in the blood (n = 3–15 mice/time point). (**C-E**) Frozen isolated human PBMCs from patients were thawed and stained with mAbs against cell-surface lineage markers for CD14^+^ monocytes (C), dendritic cells (myeloid or plasmacytoid) (D), or NK cells (E) including a viability stain, as well as indicated activation markers. Gating strategies and respective frequencies are shown. Scattered plots show the relative expression level (MFI) or percent of cell population expressing indicated marker for each individual patient at enrollment versus follow-up. (**F**) The proportion of blood Ly6C^+^ monocyte and NK cells expressing various surface markers in *Py*-infected WT mice at 1.5 and 4.5 days post infection (n = 3 = 14 mice/condition). Experiments were replicated 2–3 times. P-values are indicated when applicable.

To gain insights into the immune response associated with severe human malaria, we analyzed inflammatory mediators and immune cells in the peripheral blood of 37 Malawian children with WHO defined cerebral malaria (CM), enrolled in the Blantyre Malaria Project during the 2013 transmission season (S1A Fig). The median age for the cohort was 43 months; patients exhibited detectable parasitemia (~70x10^3^ parasites/μl), anemia (23% hematocrit) and primarily manifested retinopathy positive CM (73%). Plasma and peripheral blood mononuclear cells (PBMCs) were drawn for analysis upon admission and 30 day convalescent blood samples among survivors. Plasma levels of inflammatory cytokines (IFNα, IFNγ, TNF, IL-6) and chemokines (CCL2, CCL3, CCL4, CXCL10) were significantly higher during severe malaria compared to convalescence (Fig 1A). Using as a surrogate model of severe malaria, wild type (WT) C57BL/6 (B6) mice were inoculated with 2x10^5^ iRBCs of the lethal *Plasmodium yoelii (Py) 17X YM* strain. Mice succumbed within 5–9 days (d) post infection with very high blood parasitemia (~80% iRBC)(S1B Fig), and we report a comparable blood inflammatory mediator profile and increase to that of human patients (Fig 1B and S1A Table), consistent with the development of severe signs of malaria and subsequent death (d 4.5 and after, S1B Fig). This comparative analysis underlines a common set of elevated blood inflammatory cytokines (IFNα, IFNγ, TNF, IL-6) and chemokines (CCL2, CCL3, CXCL10) in patients and mice. Yet, the use of the mouse model further revealed an early burst (d 1.5) of blood inflammatory cytokines including type I IFN (IFNα, IFNβ) and chemokines (S1C and S1D Fig) that are likely involved in early myeloid effector cell recruitment to the blood and the initiation of systemic inflammation.

Twelve color flow-cytometry analysis on the PBMCs of these patients (S1E and S1F Fig) revealed a higher proportion of inflammatory (CD14^+^) monocytes [9, 50] and lower frequencies of pDCs [51] but no significant differences for myeloid DCs or patrolling (CD14^dim^) monocytes during CM compared to follow up (S1G Fig), a trend that was also noted in the mouse model (S1B Table). Expression of the costimulatory molecule CD86, MHC class II (MHC-II) HLA-DR and the integrin CD11c were decreased on all blood myeloid cells except on pDCs, yet the type I IFN responsive marker BST2 was always upregulated (Fig 1C and 1D and S1H and S1I Fig). Cell surface levels of the CD40 costimulatory molecule and the ICAM-1 adhesin were also increased on the various monocyte subsets. NK and T cells exhibited activated phenotypes (CD69^hi^, CD62^lo^, CD45RO^+^) and actively proliferated (Ki67^+^) (Fig 1E and S1J Fig). In *Py*-infected mice too, inflammatory (Ly6C^+^) monocytes, pDCs and NK cells underwent robust activation compared to that of uninfected controls (Fig 1F and S1K Fig). Specifically, Ly6C^+^ monocytes upregulated cell surface expression of CD86, MHC-II, CD11c, F4/80, ICAM-1 and the IFN-responsive markers BST2, Sca-1 and IL-15Rα. Increased cell surface CD86 and BST2 was also measured on pDCs, and NK cells upregulated CD69, became cytolytic (granzyme B^+^, NKG2D^+^), and secreted IFNγ. While expression of some of these markers (CD86, MHC-II, CD11c) on inflammatory monocytes (CD14^+^/Ly6C^+^) followed a different trend in the patients than in the mouse model at early time points (d 1.5), their expression was significantly down-modulated at later times (d 4.5, Fig 1F) when mice exhibited severe clinical symptoms, a physiological situation closely related to that of the patients at enrollment (S1C Table). Thus, in summary, several functional markers related to cell activation, adhesion, antigen presentation, costimulation, differentiation, proliferation, effector functions and type I IFN were strongly upregulated on the various immune cell subsets during severe malaria in human patients and in the surrogate mouse model. This is particularly noticeable for CD14^+^/Ly6C^+^ monocytes, NK cells and pDCs. The current results draw an interesting parallel between the high level and the nature of the inflammatory mediators, and the activated immune cell subsets found in the peripheral blood of patients with severe malaria and the *Py YM* surrogate mouse model.

### Type I IFN signaling promotes lethal outcomes and immune activation in severe murine malaria

**Fig 2:**
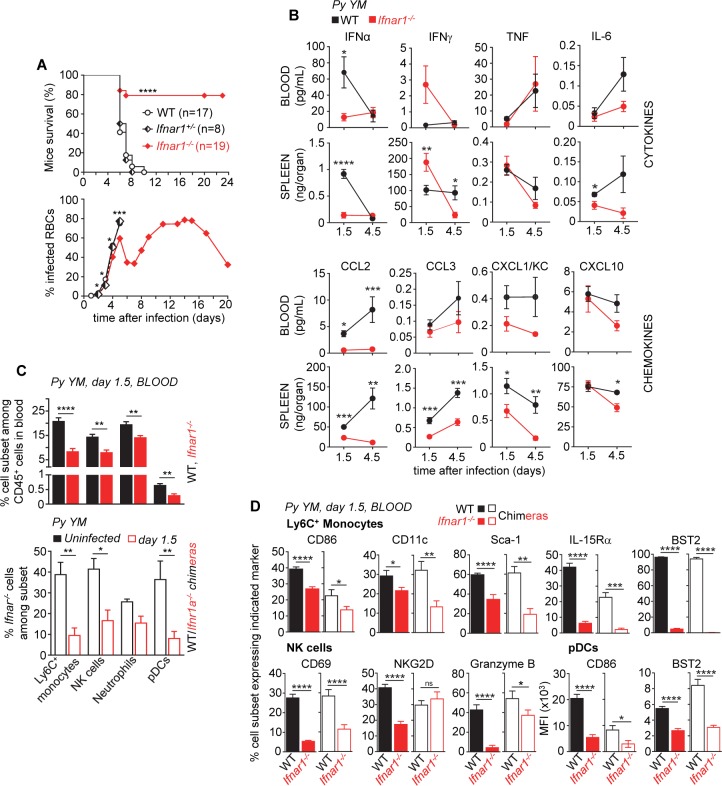
Type I interferon enhances immune blood leukocyte activation and lethal outcomes in severe murine malaria. WT or *Ifnar1* ^*-/-*^ B6 mice were inoculated i.v. with 2x10^5^ *Py 17X YM* iRBCs. (**A**) Survival and blood parasitemia of *Py*-infected mice over indicated times. (**B**) Serum and spleen cytokine and chemokine levels/contents 1.5 or 4.5 days post *Py*-infection (n = 3–8 mice). (**C**) 1.5 days after *Py* infection, blood cells were stained for the cell-surface lineage markers CD11b, CD3, CD19, Ly6C, Ly6G, NKp46, BST2, SiglecH and CD45. Frequencies of indicated cell subsets among all blood leukocytes (CD45^+^) in *Py*-infected WT compared to *Ifnar1* ^*-/-*^ mice (upper bar graph, n = 6–10 mice) and either *Py*-infected or uninfected WT CD45.1^+^/*Ifnar1* ^*-/-*^ (ratio 50:50) mixed bone marrow chimeras (lower bar graph, n = 7 mice) are shown. (**D**) The proportion of blood Ly6C^+^ monocyte and NK cells expressing various surface markers in *Py*-infected WT and *Ifnar1* ^*-/-*^ mice (n = 6–10 mice) or WT CD45.1^+^/*Ifnar1* ^*-/-*^ mixed chimeras (n = 7 mice) are reported. Experiments were replicated 2–3 times. P-values are indicated when applicable.

Since type I IFN is among the first cytokines detected (Fig 1B), and represents a key mediator of immune cell activation in human and murine malaria [16, 35–37, 41, 52], we hypothesized that induction of type I IFN may be an essential checkpoint controlling the severity of infection and associated inflammation and immune cell activation. We inoculated mice lacking the type I IFN receptor (*Ifnar1* ^*-/-*^) or control WT B6 mice with *Py YM*, and monitored survival and blood parasitemia (Fig 2A). While WT mice succumbed 5–8 d post infection, the majority of *Ifnar1* ^*-/-*^ mice survived (~80%) and controlled parasite growth, as was also reported for *Ifnar1* ^*-/-*^ mice inoculated with the *P*. *berghei ANKA* mouse model of severe malaria [35, 37]. As hypothesized, the levels of inflammatory mediators (IL-6, CCL2, CCL3, CXCL1, CXCL10 and IFNα as expected) were lower in the blood and the spleen of *Ifnar1* ^*-/-*^ mice compared to WT counterparts at all times post-infection, except for IFNγ (Fig 2B). Consistent with these findings, a significant reduction of early immune leukocyte recruitment (factor of ~1.2–3) to the blood of infected *Ifnar1* ^*-/-*^ mice was also noted, which was accounted for by direct type I IFN signaling to the various innate immune cells subsets as shown in *Ifnar1* ^*-/-*^/WT mixed chimeras (Fig 2C and S2A Fig). The activation of inflammatory monocytes (CD86^+^, CD11c^+^, Sca-1^+^, IL-15Rα^+^, BST2^+^), NK cells (CD69^+^, NKG2D^+^, Granzyme B^+^) and pDCs (CD86^+^, BST2^+^) was decreased by ~20–85% in *Ifnar1* ^*-/-*^ mice (Fig 2D, filled bars and S2B Fig). These results extended to *Ifnar1* ^*-/-*^/WT chimeras, demonstrating that direct type I IFN signaling to these cell subsets mediates their activation (Fig 2D, open bars and S2B Fig). Overall, type I IFN production and signaling during severe blood stage malaria represents a key checkpoint of inflammation and immune cell activation, particularly for inflammatory monocytes and NK cells, a finding that correlates to disease outcomes.

### Plasmacytoid dendritic cells produce immune-activating type I IFN in the bone marrow and blood of *Py*-infected mice

**Fig 3:**
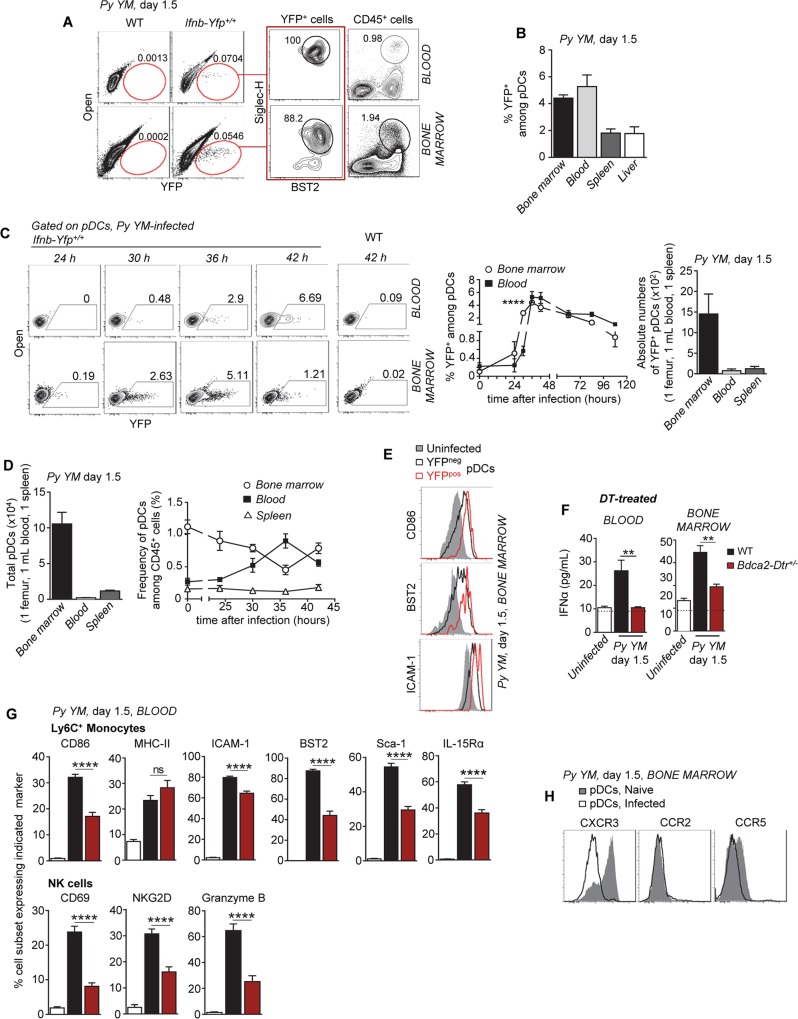
Plasmacytoid dendritic cells produce immune-activating type I IFN in the bone marrow and the blood of *Py*-infected mice. (**A**) WT *Ifnb-Yfp* ^*+/+*^ reporter mice (n>5) were inoculated i.v. with 2x10^5^ *Py 17X YM* iRBCs and blood and bone marrow cells were stained with the lineage markers CD11b, CD3, CD19, NK1.1, Ly6C, BST2 and Siglec-H. The phenotype of YFP^+^ cells is shown. (**B**) Frequencies of YFP^+^ pDCs (CD11b^lo^BST2^+^SiglecH^+^) in bone marrow, blood, spleen, and liver of *Py*-infected WT *Ifnb-Yfp* ^*+/+*^ reporter mice (1.5 day, n = 3–10 mice). (**C**) Kinetics of YFP expression by pDCs in the bone marrow and blood of *Py*-infected WT *Ifnb-Yfp* ^*+/+*^ reporter mice and absolute numbers of YFP^+^ pDCs in the indicated compartments (n = 3–11 mice/time point). (**D**) The bar graph shows the absolute number of pDCs in the bone marrow, blood, and spleen 1.5 days post infection (n = 7 mice). Kinetics of total pDC frequency among CD45^+^ cells in the bone marrow, blood, and spleen during the first 48 hours of *Py*-infection (n = 5–7 mice/time point). (**E**) Activation profiles (CD86, BST2, ICAM-1) of pDCs in the bone marrow of *Py*-infected (YFP^+^, YFP^neg^) or uninfected (YFP^neg^) *Ifnb-Yfp* ^*+/+*^ reporter mice (n>5 mice/condition). (**F, G**) DT-treated *Bdca2-Dtr* ^*+/-*^ or control WT B6 mice were inoculated with 2x10^5^ *Py 17X YM* iRBCs and 1.5 days later, levels of IFNα in the blood and bone-marrow (F, n = 7–13 mice), and activation profiles of Ly6C^+^ monocytes and NK cells using indicated markers were measured (G, n = 3–7 mice). (**H**) FACS histogram overlays of indicated chemokine receptor expression on pDCs from *Py*-infected versus naïve mice (n = 3 mice/condition). Experiments were replicated 2–4 times. P-values are indicated when applicable.

We next sought to define which cells produced type I IFN during *Py YM* infection and their tissue distribution. We utilized *Ifnb-Yfp* ^*+/+*^ reporter mice expressing the yellow fluorescent protein YFP under the control of the IFNβ promoter [53], and monitored *Ifnb* gene expression (e.g., YFP^+^ cells) by immune cells in the blood, bone marrow, spleen and liver of *Py YM* infected mice (Fig 3A and 3B). Staining of YFP^+^ cells in infected mice with cell-surface lineage markers (S3A Fig) revealed that all YFP^+^ cells in the various tissues were BST2^hi^ SiglecH^hi^ CD11b^low^ B220^int^ CD11c^int^, defining them as plasmacytoid DCs (pDCs). YFP^+^ pDCs were present predominantly in the bone marrow and blood of infected mice, with far fewer in the spleen. At least 20 times more YFP^+^ pDCs were detected in the bone marrow (factor of ~10 per femur) compared to the other compartments, which was consistent with higher levels of quantified IFNα and IFNβ (Fig 3C and S3B Fig). Thus the bone marrow contains the vast majority of IFNα/β-producing pDCs and their kinetics of appearance suggests that production of type I IFN occurs in this compartment as early as 24 hours post-infection, peaking at 36–42 hours before waning. Of note, pDC frequencies increased in the blood while decreasing in the bone marrow, consistent with the idea that following activation in the bone marrow, pDCs emigrate to the blood (Fig 3D). In line with this possibility, YFP^+^ pDCs underwent stronger upregulation of cell-surface CD86, BST2 and ICAM-1 compared to their YFP^neg^ counterparts (Fig 3E).

To further confirm that pDCs are the predominant source of type I IFN in *Py* infection, we next conducted loss of function experiments in BDCA2 depleter mice *(Bdca2-Dtr* ^*+/-*^ *)*, which express the simian diphtheria toxin receptor (DTR) under the human BDCA2 pDC-specific promoter [54]. Diphtheria toxin (DT)-treated *Bdca2-Dtr* ^*+/-*^ mice were inoculated with *Py YM*, and blood and bone marrow levels of IFNα and IFNβ were measured 1.5 d later (Fig 3F and S3C Fig). Production of both IFNα and IFNβ in these mice was abrogated compared to WT counterparts, further establishing pDCs as the major producers of systemic type I IFN during severe malaria. Selective elimination of the pDCs also impaired blood recruitment and activation of inflammatory monocytes and NK cells (Fig 3G and S3D Fig), altogether supporting the idea that pDC-derived type I IFN control immune cell activation. Yet survival of pDC-depleted mice was only slightly enhanced (S3E Fig) suggesting pDC-derived type I IFN partially contributed to disease severity in the course of severe murine *Py* malaria.

In addition, both CCR5 and CXCR3 chemotactic receptors (but not CCR2), which are expressed on mature bone marrow pDCs, and can contribute to pDC migration from the bone marrow to the blood [55, 56], underwent cell-surface downregulation in infected mice (Fig 3H and S3F Fig). Taken together, these results provide evidence that pDCs are the primary detectable cell type responsible for secretion of systemic type I IFN during severe malaria, with the bulk of production occurring in the bone marrow (also likely the site of initial activation, See kinetics Fig 3C) and in the blood.

### MyD88 and STING control systemic type I IFN production during severe malaria

**Fig 4:**
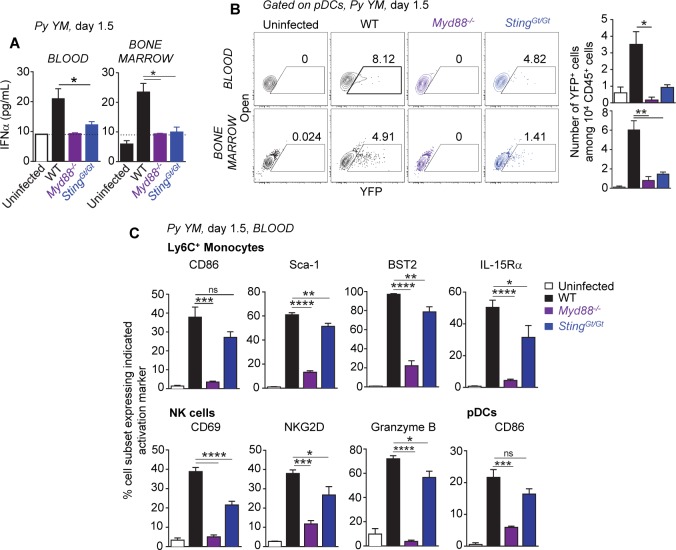
Production of systemic type I IFN during severe murine blood stage malaria requires both MyD88 and STING sensing pathways. WT, *Myd88* ^*-/-*^ or *Sting* ^*Gt/Gt*^ B6 mice crossed to *Ifnb-Yfp* ^*+/+*^ reporter mice were inoculated i.v. with 2x10^5^ *Py 17X YM* iRBCs. 1.5 days later, levels of IFNα (**A,** n = 4–10 mice/genotype) and frequencies of YFP^+^ cells among pDCs, as well as absolute numbers (**B**), in the blood and bone marrow were determined (n = 3–15 mice/genotype). (**C**) Blood cells were stained for the cell-surface lineage markers CD11b, Ly6C, NKp46, BST2, Siglec-H, CD45, and indicated activation markers. Expression of activation markers on Ly6C^+^ monocytes, NK cells and pDCs in the blood of *Py*-infected compared to uninfected mice is shown (n = 3–8 mice/genotype). Experiments were replicated 2–3 times. P-values are indicated when applicable.

Both the MyD88 and the STING sensing pathways may contribute to type I IFN production in response to *Plasmodium* parasite-derived molecules [35, 36, 41]. To define whether and to which extent any of these sensing pathways control systemic type I IFN secretion during severe malaria *in vivo*, we first inoculated *Myd88* ^*-/-*^, *Sting* ^*Gt/Gt*^ or control WT mice with *Py YM*, and measured blood and bone marrow IFNα/β levels at its peak (d 1.5) of production (Fig 4A and S4A Fig). While as reported earlier, a significant increase in IFNα and IFNβ was detected in the blood of *Py*-infected WT mice, only levels close to that of uninfected mice, i.e., at the limit of detection of the assay, could be measured in *Myd88* ^*-/-*^ and *Sting* ^*Gt/Gt*^ mice. We next infected the *Ifnb-Yfp* ^*+/+*^ reporter mice crossed to either *Myd88* ^*-/-*^, *Sting* ^*Gt/Gt*^ or control WT mice, and monitored expression of the *Ifnb* gene, e.g., YFP^+^ cells, by immune cells -all YFP^+^ cells were pDCs- in the blood, bone marrow and spleen (Fig 4B and S4B Fig). In line with IFNα/β protein levels, YFP^+^ pDCs were undetectable in *Myd88* ^*-/-*^ *Ifnb-Yfp* ^*+/+*^ mice and reduced by over two-third in *Sting* ^*Gt/Gt*^ *Ifnb-Yfp* ^*+/+*^ mice, compared to WT controls. Consistent with the role of type I IFN in activating the immune system during *Py* infection (Fig 2), the recruitment and maturation of blood Ly6C^+^ monocytes, NK cells and pDCs, was strongly impaired in *Myd88* ^*-/-*^ mice (Figs 4C and S4C). These differences remained modest in *Sting* ^*Gt/Gt*^ compared to WT mice, suggesting either that the low frequency of IFNβ-expressing pDCs (YFP^+^) -and likely of secreted IFNα- produced in *Py*-infected *Sting* ^*Gt/Gt*^ mice was sufficient to induce immune cell activation or that other, type I IFN-independent STING-dependent signals, influence this process. Altogether, these results establish the importance of both MyD88 and STING sensing pathways for systemic pDC-derived type I IFN production during severe blood stage malaria.

### Plasmacytoid dendritic cells produce type I IFN via TLR7/MyD88 during malaria *in vivo*

**Fig 5:**
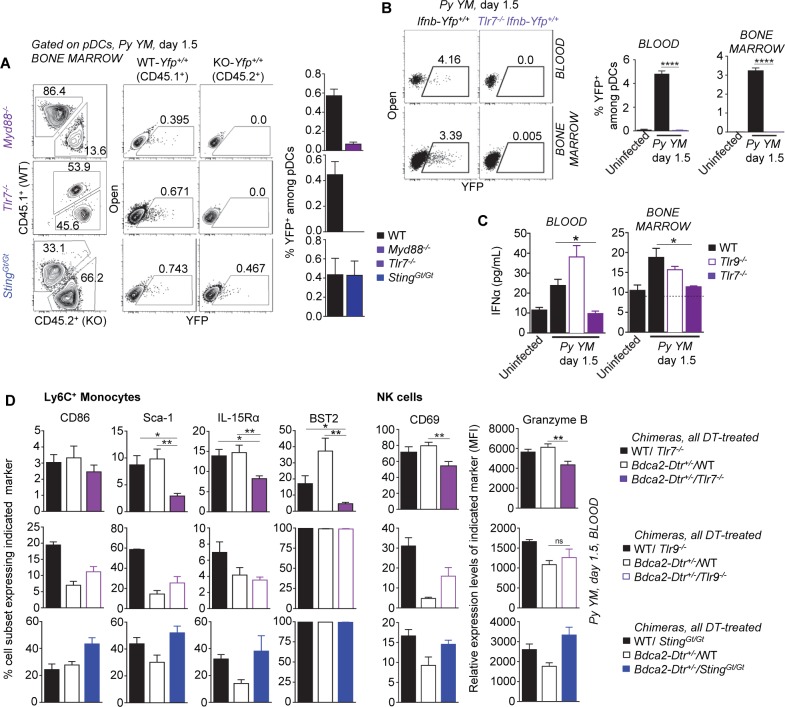
Plasmacytoid dendritic cells produce immune-activating type I IFN via TLR7/MyD88 but not STING. (**A**) Frequencies of WT (CD45.1^+^) or KO (*Myd88* ^*-/-*^, *Tlr7* ^*-/-*^, *Sting* ^*Gt/Gt*^) YFP^+^ pDCs in the bone marrow of *Py*-infected WT/KO *Ifnb-Yfp* ^*+/+*^ mixed chimeras (n = 4–6 mice/chimera). Bar graphs average all individual mice across 2 replicate experiments. (**B**) Frequency of YFP^+^ pDCs in the bone marrow of WT and *Tlr7* ^-/-^ *Ifnb-Yfp* ^*+/+*^ reporter mice 1.5 day post *Py* infection (n = 4 mice/genotype). Bar graphs summarize the frequencies of YFP^+^ pDCs in the blood and bone marrow. (**C**) IFNα levels in the blood and the bone marrow of *Py*-infected *Tlr7* ^*-/-*^, *Tlr9* ^*-/-*^, WT mice or uninfected mice (n = 4–8 mice/genotype). **(D)** *Bdca2-Dtr* ^*+/-*^/*Tlr7* ^*-/-*^, *Bdca2-Dtr* ^*+/-*^/*Tlr9* ^*-/-*^, *Bdca2-Dtr* ^*+/-*^/*Sting* ^*Gt/Gt*^ or control WT/KO (ratios 50:50 or 30:70, see S5 Fig) mixed bone marrow chimeras (n = 3–5 mice/chimera) were treated with DT prior *Py* infection, and activation profiles of blood Ly6C^+^ monocytes and NK cells in the blood using indicated markers were measured. Experiments were replicated 2–4 times. P-values are indicated when applicable.

MyD88 and STING, and pDCs accounted for systemic type I IFN production in severe malaria (Fig 4). To determine whether either pathway is active in pDCs, we generated mixed bone marrow chimeras reconstituted with either *Myd88* ^*-/-*^ or *Sting* ^*Gt/Gt*^ and WT donor cells, all expressing the *Ifnb-Yfp* ^*+/+*^ reporter transgene, and monitored the presence of YFP^+^ pDCs following *Py*-infection (Fig 5). Lack of MyD88 but not STING in pDCs abrogated transcription of the *Ifnb* gene in *Py*-infected chimeras (Fig 5A). Since TLR7 and TLR9 are functionally active in pDCs upstream of MyD88 [57], we tested their implication in MyD88-dependent production of type I IFN. YFP^+^ *Tlr7* ^*-/-*^ pDCs were neither detected in *Tlr7* ^*-/-*^/WT *Ifn*β*-Yfp* ^*+/+*^ mixed chimeras nor in *Tlr7* ^*-/-*^ *Ifnb-Yfp* ^*+/+*^ mice, further establishing that lack of TLR7 in pDCs fully prevents *Ifnb* gene expression (Fig 5A and 5B). Likewise, production of IFNα in the blood and bone marrow of *Py*-infected *Tlr7* ^*-/-*^ mice was largely abrogated compared to WT mice (Fig 5C). Unexpectedly [31, 33, 34], we did not detect any significant differences in IFNα levels in the blood or bone marrow of *Py*-infected *Tlr9* ^*-/-*^ mice, ruling out a role for TLR9 in this model. Of note, the frequency of YFP^+^ pDCs and associated levels of type I IFN, were consistently lower in the chimera mouse models compared to non-irradiated counterparts (See Fig 3C versus Fig 5A).

To further link pDC production of type I IFN via these pathways to immune cell activation, we generated additional mixed chimeras reconstituted with *Bdca2-Dtr* ^*+/-*^ and either *Tlr7* ^*-/-*^, *Tlr9* ^*-/-*^ or *Sting* ^*Gt/Gt*^ bone marrow (Fig 5D and S5 Fig). In these mice, WT pDCs are eliminated following DT injection while pDCs lacking either of the above pathways remain. As predicted from type I IFN levels (Figs 4A and 5C), blood inflammatory monocyte and NK cell activation were impaired when pDCs lacked TLR7 but not TLR9 or STING (Fig 5D). Altogether, these data establish pDCs as key producers of type I IFN in the course of severe malaria, through the TLR7/MyD88 pathway. Moreover, pDC-secreted type I IFN directly promoted immune cell activation that correlates with disease severity.

### CD169^+^ macrophages prime pDCs to secrete type I IFN

**Fig 6:**
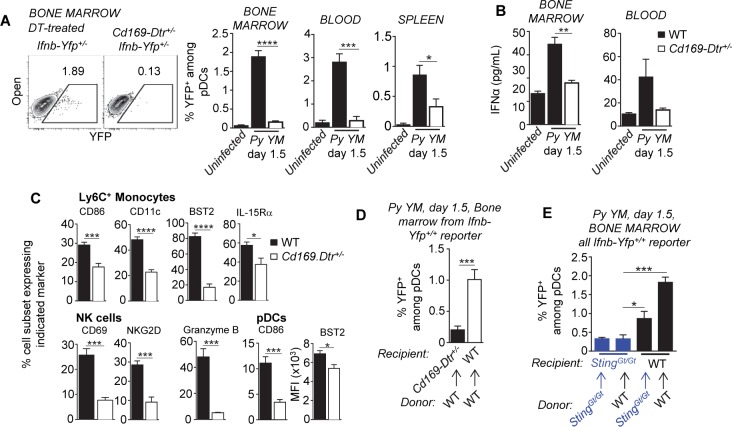
CD169^+^ macrophages control early pDC activation in the bone marrow of infected mice. (**A**) DT-treated WT and *Cd169-Dtr* ^*+/-*^ *Ifnb-Yfp* ^*+/+*^reporter mice (n = 7–14 mice/genotype) were inoculated i.v. with 2x10^5^ *Py 17X YM* iRBCs and blood and bone marrow cells were stained with the lineage markers CD11b, BST2 and Siglec-H and frequencies of YFP^+^ pDCs is shown. In (**B**), levels of IFNα in the blood and bone marrow of these mice were quantified and (**C**) shows the activation profiles of Ly6C^+^ monocytes, NK cells and pDCs using indicated markers (n = 3–7 mice/condition). (**D**) WT or *Cd169-Dtr* ^*+/-*^ recipient mice (n = 7 mice/chimera) reconstituted with bone-marrow cells from WT *Ifnb-Yfp* ^*+/+*^ mice were DT-treated and frequencies of YFP^+^ pDCs in the bone marrow was determined 1.5 days post *Py* infection. (**E**) Frequencies of YFP^+^ pDCs in the bone marrow of indicated *Py*-infected WT or *Sting* ^*Gt/Gt*^ reciprocal chimeras n = 4 mice/chimera). Experiments were replicated 2–4 times. P-values are indicated when applicable.

STING contributes to systemic type I IFN production during *Py* infection, but this is extrinsic to pDCs (Figs 4 and 5A). Prior work in human macrophage cell lines proposed that STING senses AT-rich stem-loop motifs from *Plasmodium* parasite DNA [35], therefore we hypothesized that STING triggering inside macrophages may provide low levels of type I IFN that could "prime" pDCs to further secrete high amounts of type I IFN. We used CD169 depleter mice in which tissue-resident CD169^+^ macrophages (F4/80^hi^) can be selectively eliminated after DT injection [58], and inoculated DT-treated *Cd169-Dtr* ^*+/-*^ *Ifnb-Yfp* ^*+/+*^ or control WT *Ifnb-Yfp* ^*+/+*^ reporter mice with *Py YM* (Fig 6A and 6B). Only very low frequencies of YFP^+^ pDCs were detected in the blood, bone marrow and spleen of DT-treated CD169 depleter mice, and correspondingly low levels of IFNα, demonstrating that CD169^+^ macrophages were required for pDCs to produce type I IFN during this infection. Of note, total pDC frequencies remained either comparable or increased (bone marrow) in DT-treated versus untreated *Cd169-Dtr* ^*+/-*^ mice, ruling out direct depletion of pDCs in this model (S6A Fig). In line with our prior findings, elimination of CD169^+^ macrophages also prevented blood immune cell activation, specifically that of Ly6C^+^ monocytes, NK cells and pDCs (Fig 6C). We confirmed these results using clodronate liposome-treated *Ifnb-Yfp* ^*+/+*^ mice, and DT-treated *LysM-Cre* ^*+/-*^ *Rosa26-iDtr* ^*+/-*^ mice, as different means to deplete tissue-resident macrophages (S6B and S6C Fig).

CD169^+^ macrophages were reported to be largely radio-resistant [59]. Consistent with this fact, the production of IFNβ by pDCs (YFP^+^) was abrogated in *Py*-infected DT-treated *Cd169-Dtr* ^*+/-*^ but not WT mice reconstituted with *Ifnb-Yfp* ^*+/+*^ WT bone marrow cells (Fig 6D). Thus the majority of IFNβ-producing DCs (YFP^+^) (>75%) undergo "priming" by radio-resistant CD169^+^ macrophages. To further link STING-mediated activation and CD169^+^ macrophages, we next asked whether STING in the radio-resistant compartment was essential to promote type I IFN production by pDCs. WT or *Sting* ^*Gt/Gt*^ mice were lethally irradiated and reconstituted with either *Sting* ^*Gt/Gt*^ or WT *Ifnb-Yfp* ^*+/+*^ reporter bone-marrow cells, and the presence of YFP^+^ pDCs was monitored 1.5 d post *Py YM* infection (Fig 6E). As hypothesized, STING inside the radio-resistant compartment was required for pDCs to secrete type I IFN. STING is a key inducer of type I IFN [60], and consistent with the idea that STING-dependent production of type I IFN is an essential orchestrator of pDC "priming", *Ifnar1* ^*-/-*^ pDCs exhibited impaired activation compared to WT pDCs (Fig 2D). Thus collectively these results support a model in which STING-activated CD169^+^ macrophages prime pDCs -possibly through very low levels of secreted type I IFN- which synergizes with pDC-intrinsic TLR7/MyD88-mediated activation.

### pDCs and CD169^+^ macrophages interact in the bone marrow of malaria-infected mice

**Fig 7:**
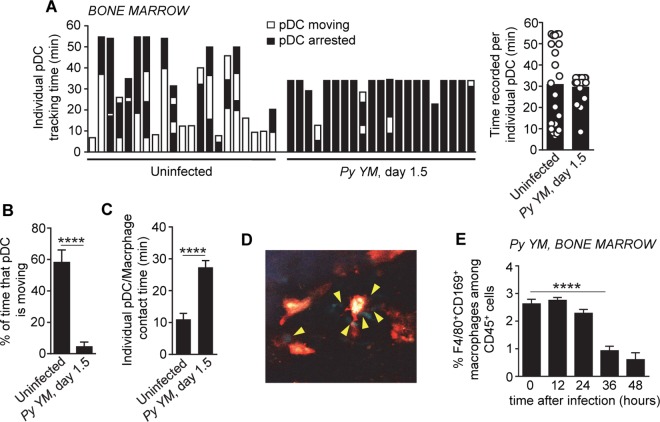
CD169^+^ macrophages form stable long-lasting interactions with pDCs in the bone marrow of *P*. *yoelii*-infected mice. (**A**) Tracking time in which individual pDCs were either moving or arrested in the bone marrow among total time monitored using intravital microscopy in *Py-infected* or uninfected live *Ptcra-Gfp* ^*+/+*^ mice (n = 5 mice/condition). Bar graph summarizes the tracking time (30″/frame) recorded per individual pDC during intravital microscopy of the bone marrow. Each open symbol on the bar graph corresponds to individual pDCs. (**B**) Bar graph summarizes the percentage of time in which individual pDCs were moving among total time monitored by intravital microscopy of the bone marrow. (**C**) Total contact time of pDCs arrested over observation time. (**D**) Frame of an intravital microscopy movie showing pDCs (green, highlighted with yellow arrows) interacting with CD169^+^ macrophages (red). **(E)** Frequency of F4/80^hi^CD169^+^ macrophages detected in the bone-marrow at various times post *Py* infection (n = 5 mice). P-values are indicated when applicable.

**Fig 8:**
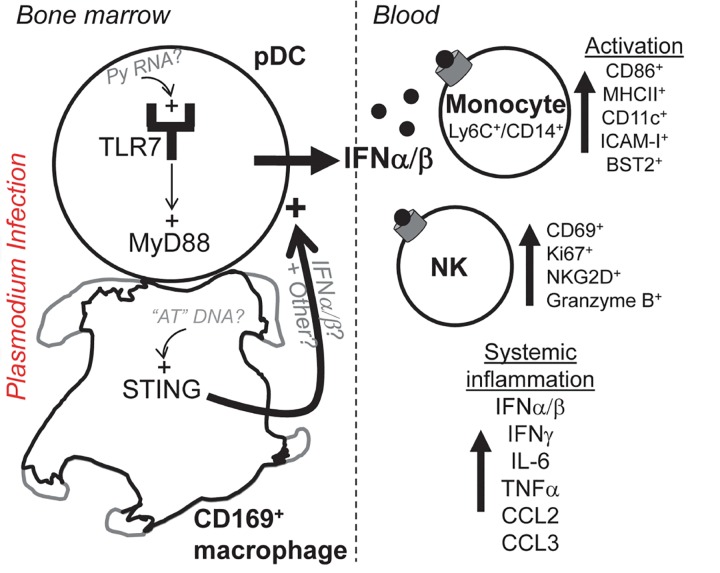
Mechanistic model summarizing findings.

Since CD169^+^ macrophages control pDC activation, we then asked whether pDCs interacted with CD169^+^ macrophages in the bone marrow. We used intravital time-lapse two-photon imaging to visualize pDCs and CD169^+^ macrophages located within the tibia of live, *Py*-infected or control uninfected mice. Taking advantage of *Ptcra-Gfp* ^*+*^ reporter mice [61] in which GFP is expressed almost exclusively by bone marrow pDCs (>95%), we injected fluorescently-labeled (phycoerythrin, PE) anti-CD169 mAb allowing for tracking of both pDCs and CD169^+^ macrophages *in vivo* (Fig 7A–7D and S1 and S2 Movies). In uninfected mice, pDC exhibited a surveying and "hopping" behavior, with transient interactions with CD169^+^ macrophages. In stark contrast, the vast majority of pDCs in infected mice arrested (Fig 7A and 7B), maintained sustained interactions (Fig 7C) and elongated and clustered with CD169^+^ macrophages (Fig 7D). This latter behavior was also observed for YFP^+^ pDCs in *Py*-infected *Ifnb-Yfp* ^*+/+*^ mice (S3 Movie). Interestingly, the majority of CD169^+^ macrophages were lost between 24 to 36 hours post infection (Fig 7E), which correlates with the kinetics of type I IFN production by pDCs, and supports the idea that macrophages may die and release their content to activate pDCs. In line with this possibility, we also observed lower numbers of CD169^+^ macrophages in the bone marrow of *Py*-infected mice (1.5 d) by intravital imaging. The remaining CD169^+^ macrophages adopted a different morphology, were less "dendritic" in shape and we noted increased PE^+^ cell debris, consistent with cell death (S1, S2 and S3 Movies). Thus, in the course of *Py* infection, pDCs arrested, clustered and contacted CD169^+^ bone marrow macrophages for prolonged periods of time, a result supporting the idea that CD169^+^ macrophages license pDCs to produce high levels of type I IFN, possibly via STING-induced type I IFN and/or releasing intracellular materials (Fig 8).

## Discussion

This study reveals novel cellular and molecular mechanisms that control the initiation of the early immune response during severe malaria infection. We characterized a highly activated and inflammatory state using a mouse model that recapitulates features of the immune response found in humans with severe malaria, and we identify broad and robust activation of multiple immune cell populations. We reveal that the antiviral cytokine type I IFN is a major orchestrator of these processes during blood stage *Plasmodium* infection, leading to enhanced inflammatory (Ly6C^hi^/CD14^+^) monocyte and NK cell activation in the blood of infected mice. We establish that pDCs produce most of the systemic immune activating type I IFN in both the bone marrow and the blood of infected mice, following TLR7/MyD88-sensing of *Plasmodium* parasites. pDCs only secreted type I IFN upon CD169^+^ macrophage “priming” in the bone marrow of infected mice. Macrophage licensing of pDCs requires STING sensing of parasites and both cell subsets establish stable long-lasting interactions, as visualized by intravital microscopy. Altogether, our data suggest a model in which STING-activated CD169^+^ macrophages may deliver low levels of type I IFN and release parasite RNA to interacting pDCs in the bone marrow of malaria-infected mice, leading to the massive production of type I IFN by the pDCs.

Severe infection with *P*. *falciparum* continues to result in high morbidity and mortality, despite robust antimalarial therapy. The clinical manifestations and pathology are well described and field studies have shown that severe malaria is associated with high inflammation, yet the mechanisms of immune dysregulation resulting in such high inflammation remain poorly understood. Adjunct measures to reduce the inflammatory syndrome and improve outcomes in severe malaria have been unsuccessful to date, and thus further studies to define the basis of immune dysregulation are needed [62]. In this work, we provide a comprehensive characterization of immune cells and inflammatory status in a cohort of pediatric severe malaria patients. We report high plasma levels of proinflammatory cytokines and chemokines (IFNγ, TNF, IL-6, CCL2, CCL3, CCL4, CXCL10) in severe malaria patients compared to baseline uninfected follow-up, which matched that of *Py* infection in mice in which we could conduct a temporal analysis. Only few studies have performed functional immunophenotyping during severe human malaria [21, 25], and most of them focused on specific subsets of immune cells, namely NK cells, γδT cells and CD4^+^ T cells. Our study examined both myeloid and lymphoid cell compartments to provide a global analysis of blood immune leukocytes during severe human malaria. Data reveal both expansion and activation of inflammatory monocytes (CD14^+^), which express an exquisite machinery to sense pathogen-derived molecules, produce inflammatory cytokines and contribute to systemic inflammation [63]. We also found diminished pDC blood frequencies in severe malaria patients as reported before [51], yet our data further reveal that pDCs were activated. pDCs represent by far the major type I IFN-producing cells *in vivo* [57] in response to viral infections, during autoimmunity and even bacterial infection, through TLR7/ and TLR9/MyD88 sensing of nucleic acids. pDCs are capable of secreting type I IFN in response to *Pf* schizonts *in vitro* [51] and they can produce type I IFN in the *P*. *chabaudi* mouse model [36], both results consistent with our data.

While NK cell blood proportions did not differ in acutely infected versus follow-up patients, these cells were strongly activated in severe malaria patients and in the *Py* mouse model, in line with prior *in vitro* reports [64–66]. Likewise and as also suggested before [67], a slight decrease in the blood proportion of CD8^+^ and CD4^+^ T cells was observed in severe malaria patients and both subsets of T lymphocytes underwent robust activation, proliferation and differentiation.

Collectively, our data draw a parallel between the immune status of a cohort of patients with severe malaria and the surrogate *P*. *yoelii 17X YM* mouse model. Blood immune cells, notably CCR2^+^ monocytes, NK cells and pDCs underwent robust activation and common inflammatory mediators were noted. A similar expression pattern of cell-surface activation markers between severe malaria patients and the mouse model could be found, notably at later time points (d 4.5) post infection. While this remains speculative, it may suggest that the initiating events driving severe immune activation and inflammation, and poor clinical outcomes are comparable to that of human patients.

Recent reports have dissected fine sensing mechanisms of *Plasmodium* parasites. At liver stage, parasite RNA is detected by the cytosolic sensor Mda5 inside hepatocytes, which initiates the host immune response [68]. During blood stage, TLR7 via MyD88 may act as an early sensor of several *Plasmodium* species [41]. *In vitro* data have also reported that "AT-rich" stem loop motifs of *Plasmodium* DNA are recognized via the STING cytosolic pathway [35]. The current results make a link between these findings, and reveal the relative importance of both TLR7/MyD88 and STING sensing pathways during severe malaria infection *in vivo*, and in which cell subsets they act. We provide novel evidence that the bone marrow, a site undergoing major functional changes during this infection [48, 69], may also represent an important site of initiation of the innate immune response against this parasite, a finding that has not been previously documented since most reports have focused on the spleen, and to some extent the blood. Intravital imaging of pDCs and CD169^+^ macrophages directly in the bone marrow of infected mice -which we are the first to report- further supports this interpretation. While pDCs exhibit a surveying and "hopping" type of behavior from one CD169^+^ macrophage to another in bone marrow and at steady state, their arrest and prolonged clustering around the macrophages after the infection are reminiscent to that of T or NK T cells after they “see” their cognate antigen [70, 71].

Since both CD169^+^ macrophages and the STING pathways in the radio-resistant compartment are required to activate the type I IFN response, we concluded that STING exerts its function inside CD169^+^ macrophages. Such interpretation is consistent with STING sensing of *Plasmodium* AT-rich motifs in macrophage cell lines *in vitro* [35]. A major consequence of STING triggering leads to the production of type I IFN [60] and although we could not detect YFP^+^ CD169^+^ macrophages in *Ifnb-Yfp* ^*+*^ reporter mice, it is likely that low levels of CD169^+^ macrophage-derived type I IFN in the bone marrow is among key signals to "prime" pDCs. Given that levels of IFNα/β are comparably reduced in *Sting* ^*Gt/Gt*^ and in *Myd88* ^*-/-*^ mice, it may also be that other, non-type I IFN dependent signals downstream of STING may drive immune cell activation.

pDCs also need to recognize parasites in a TLR7/MyD88-dependent manner in order to produce type I IFN, which suggests the requirement for several signals -at least two- to achieve their full type I IFN-producing capacity, through cell-intrinsic TLR7 and cell-extrinsic STING. Selective depletion of CD169^+^ macrophages largely prevents subsequent activation of immune effector cells while lacking STING only partially impairs (by ~20–50%) immune activation. One likely possibility is that macrophages provide other signals to the pDCs, notably other non-RNA parasite-derived (or not) ligands and/or cytokines. Macrophages can scavenge iRBCs and may undergo subsequent activation and pyroptotis [34], ultimately releasing their cytosolic contents to interacting pDCs. The concomitant loss of CD169^+^ macrophages with their morphological changes observed by intravital microscopy in the bone marrow between 24 and 36 hours post *Py* infection is consistent with such interpretation. Another non-exclusive possibility could be that macrophages deliver signals to pDCs by secretion of exosomes-containing parasite RNA. pDCs may indeed be using such indirect mechanisms of activation as suggested in the case of viral infections [72–74].

The model we favor proposes that pDCs produce systemic type I IFN which orchestrates immune activation and contributes to deleterious outcomes during lethal malaria. Yet while selective depletion of pDCs prevents massive immune cell activation *in vivo*, it only has a modest impact on mice survival contrary to that of *Ifnar1* ^*-/-*^ mice, suggesting that low levels of type I IFN may be produced and consumed locally by many other cell types. Disrupting type I IFN signaling is also known to promote enhanced IFNγ production [75], a cytokine exhibiting protective effects during malaria [76], and this may account for these seemingly discrepant findings. Another possible interpretation is that IFNβ, in contrast to all other IFNα isoforms, have a unique non-Jak/STAT pathological signaling feature through IFNAR1, independent of IFNAR2 (the high affinity receptor for IFNα/β) as reported in a model of LPS-induced sepsis [77]. If true, disrupting IFNAR2 should not phenocopy the survival advantage of *Ifnar1* ^*-/-*^ mice but that of the WT counterparts. Thus clearly, further investigations will be needed to resolve these important issues in future work.

An additional intriguing difference with regards to current literature relates to the *P*. *berghei* murine model of CM, in which type I IFN signaling to DCs was shown to prevent Ly6C^hi^ monocyte and cDC activation, and subsequent optimal priming of protective Th1 CD4^+^ T cell responses while its disruption enabled better control of parasite infection and host survival [37]. Despite similar outcomes, the proposed mechanisms have to be very different, which is likely accounted for by the use of very distinct surrogate models of severe malaria in which host resistance or death are not regulated by the same processes. For instance, in this model and in contrast to *Py YM* infection, the burst of systemic type I IFN occurs much later (day 4 versus day 1.5), parasitemia stay very low (1.5% versus ~60% iRBCs by day 4), and significant differences in MHC class II expression on WT versus *Ifnar1* ^*-/-*^ monocytes are reported, altogether supporting the idea that immune cells are exposed to very different milieus both at early and late times post infection. Yet, these results overall underline the importance and the need for further investigations of the role of type I IFN in host resistance during malaria.

## Materials and Methods

### Human studies

#### Patient population

To characterize immune responses associated with severe human malaria we examined Malawian children with CM, enrolled in the Blantyre Malaria Research Project (BMP), an ongoing longitudinal study of CM, during the 2013 transmission season [78]. Children between the ages of 6 months and 12 years with *P*.*falciparum* infection and a Blantyre Coma Score <3 and parental consent were enrolled, and those with a positive blood or CSF bacterial culture were excluded. Whole blood samples were collected at enrollment into the BMP, and at 30 days after discharge. A fundoscopic exam was performed to determine the presence (Ret^+^CM) or absence (Ret^-^CM) of malarial retinopathy [78]. Clinical characteristics and laboratory data were extracted from the study database.

#### Sample preparation

Six mLs of blood was collected from enrolled patients, and PBMCs were isolated using Ficoll separation within 6 hours of blood draw. Isolated PBMCs were resuspended in FCS/10% DMSO and immediately transferred to a Mr. Frosty freezing container and put in -80°C freezer for 24 hours, before being transferred and stored in liquid nitrogen and shipped to Albert Einstein College of Medicine for analysis.

### Mice

Wild-type (WT) C57BL/6J (B6) 6–12 wk-old male mice, congenic CD45.1^+^ (strain 2014, Jackson Labs), *Ifnar1* ^*-/-*^ [79] (Kind gift Jake Kohlmeier, Emory Vaccine Center), *Ifnb* ^*mob/mob*^ */ Ifnb-yfp* ^*+/+*^ [53] (strain 10818, Jackson Labs), *Sting* ^*Gt/Gt*^ [80] (strain 17537, Jackson Labs), *Tlr7* ^*-/-*^ [81] (strain 8380, Jackson Labs), *Tlr9* ^*-/-*^ [82] (kind gift Alice Prince, Columbia University), *Myd88* ^*-/-*^ [83] (strain 9088, Jackson Labs), *Bdca2-Dtr* ^*+/-*^ [54] (strain 14176, Jackson Labs), *Cd169-Dtr* ^*+/-*^ [58, 84] (kind gift Masato Tanaka, Riken) and *Ptcra-Gfp* ^*+/+*^ [61, 85] were housed and bred in our SPF animal facility for all experiments (AECOM).

### Ethic Statements

#### Human research

IRB approvals were obtained from the Albert Einstein College of Medicine, Michigan State University and The University of Malawi College of Medicine Research and Ethics Committee. Written informed consent from the parent or guardian of any child participant was obtained on their behalf.

#### Animal research

This study was carried out in strict accordance with the recommendations by the animal use committees at the Albert Einstein College of Medicine. The institution is accredited by the "American Association for the Use of Laboratory Animals" (DHEW Publication No. (NIH) 78–23, Revised 1978), and accepts as mandatory the NIH "Principles for the Use of Animals". All efforts were made to minimize suffering and provide humane treatment to the animals included in the study. Approved study protocol is 20141202.

### Generation of bone marrow chimera mice

WT B6 mice received a lethal dose of irradiation (1,200 rads), and were immediately reconstituted with 1-2x10^6^ bone marrow cells isolated from flushed femurs from indicated donor mice, and antibiotic treated for 2 weeks. Reconstituting target ratio (post irradiation) and actual ratio (post-reconstitution in blood and spleen, 6–8 weeks later) of bone marrow donor cells are specified for each experiment legend and associated supplementary figures. We usually determined mixed chimera ratios to minimize the impact of cell-specific depletion and of having too high a proportion of knockout versus WT hematopoietic-derived cells (like in the case of WT/*Myd88* ^*-/-*^ chimeras).

### *Plasmodium* infections, measure of blood parasitemia and mice survival

#### Infections

*Plasmodium yoelii (17X)*, *clone YM* parasites (stock MRA-755) were obtained from the Malaria Research and Reference Reagent Resource Center as part of the BEI Resources Repository, NIAID, NIH (Manassas, VA; Deposited by D. Walliker). *P*. *yoelii YM*-infected red blood cells (iRBCs) from a frozen stock (stored in liquid nitrogen in Alsever’s solution and 10% glycerol) were intraperitoneally (i.p.) injected into one WT B6 mouse and grown for ~4 days. When parasitemia reached 2–5%, 2x10^5^ *P*. *yoelii YM* iRBCs were infected intravenously into each experimental mouse, unless otherwise indicated.

#### Parasitemia

Blood parasitemia was determined by flow cytometry as described [86]. One μL of blood was obtained by cutting the tip of the tail with a sterile razor, and blood was deposited in 200μl of 0.025% glutaraldehyde in phosphate buffered saline (PBS) containing 1mM EDTA in a 96-well plate. Fixed red blood cells were washed with PBS and then permeabilized with 0.25% Triton X-100 (Sigma) in PBS for 5 min. After centrifugation, red blood cells were incubated in 1mg/mL RNAse A (Sigma) for 30 min at room temperature. Red blood cells were then stained with 0.5 μM of the YOYO-1 dye (Invitrogen) for 30 min at room temperature and directly analyzed on a BD FACSCanto II (Becton Dickinson, CA), equipped with a 488-nm argon laser, using a 530/30 emission filter to detect YOYO-1 fluorescence. Red blood cells were gated based on forward and side scatter, and parasitemia was determined as the frequency of YOYO-1 positive cells among all cells. Eye-counting of Giemsa-stained blood smears by microscopy confirmed that determination of parasitemia using the above method gave comparable results.

#### Mice survival

Mice were monitored for survival twice a day after day 4, in the morning and evening.

### Cell-specific depletion *in vivo*

Depleter mice, e.g., *Cd169-Dtr* ^*+/-*^, *Bdca2-Dtr* ^*+/-*^, *LysM-Cre-iDtr* ^*+/-*^ as well as mixed depleter bone-marrow chimeras (as specified in relevant figures), received 10ng/g body weight of diphtheria toxin (DT, Calbiochem) intraperitoneally (i.p.), 12 hours before *Plasmodium* infection unless indicated otherwise. For clodronate liposomes (Clodrosome), mice were injected i.v. with 250 μl of clodronate-loaded or control liposomes 12 hours prior infection.

### Preparation of cell suspensions for flow cytometry (FACS) analysis

Spleens were dissociated on nylon meshes (100μm) and incubated at 37°C for 20 min in HBSS medium (Gibco) containing 4,000 U/ml of collagenase I (Gibco) and 0.1 mg/ml of DNase I (Roche), and red blood cells (RBC) further lysed with NH_4_Cl buffer (0.83% vol/vol). Blood was harvested into heparin tubes and red blood cells lysed as above. Cell suspensions were further used for the different analyses (see below). Bone marrow was harvested from the femur by cutting off the ends of the femur, centrifuging the femur at 16000 rcf using an Eppendorf bench top centrifuge, and resuspending the resulting cell pellet in media, mixing until a single cell suspension was achieved.

### Cell staining for FACS analysis

#### Mice

Cell suspensions prepared as above were incubated with 2.4G2 antibody for 15 min on ice and further stained with specified antibodies cocktail (See **S1 Table**) in FACS buffer (PBS 1% FCS, 2mM EDTA, 0.02% sodium azide). For intracellular IFNγ staining, cells were incubated for 3–4 hrs at 37°C, 5% CO_2_ in RPMI 1640 (Invitrogen) 5% FCS, Golgi Plug (BD), fixed in IC fixation buffer (eBioscience) for 15 min at 4°C, and permeabilized for 30 min in 1X Perm/Wash buffer (eBioscience) containing indicated intracellular marker(s). For intracellular Granzyme B staining, unstimulated cells were fixed with Foxp3/Transcription Factor Fixation/Permeabilization Buffer (eBioscience) for 15 min at 4°C, and permeabilized for 30 min in 1X Perm/Wash buffer (eBioscience) containing anti-Granzyme B. For determining absolute numbers of cells, we have made use of counting beads (SpheroTech) as internal standard added in cells isolated from either one femur or one spleen (20,000 beads) or from a given volume of blood (3960 beads for 120 μl of blood).

#### Human

Frozen Ficoll-separated human PBMCs (in FCS/10% DMSO) were thawed quickly in a 37°C water bath, until only a small ice pellet remained, transferred to 12mLs of room temperature RPMI 1640 (Invitrogen) 5% FCS, and immediately washed. Cells were then resuspended in FACS buffer for staining with specified antibody combinations. Intracellular Tbet and Ki67 staining were done as described for Granzyme B. Stained cells were collected either on a FACS BD LSR-II or Aria III. A list of all mAbs and sera used in the study with full information is provided in **S1 Table.** Data were analyzed using FlowJo version 9.6.2 (TriStar) on G5 Macintosh computers.

### Measure of cytokine and chemokine contents

#### Mice

Spleens from immunized mice were harvested, snap-frozen and stored at -80°C until further processing. Spleens were then thawed in 150μl of lysis buffer (150mM NaCl, 40mM Tris pH 7.4) containing a 1mM PMSF and a cocktail of protease inhibitors (Promega), homogenized with a douncer, and supernatant frozen at -80°C, thawed and centrifuged to obtain cleared supernatants. For bone marrow, content was harvested as described above for FACS analysis, resuspended in 150μl of lysis buffer, centrifuged, and supernatant was frozen at -80°C. Serum was obtained by collecting blood in a unheparanized tube, allowing clotting for 15 minutes at room temperature before centrifugation for 20 min at 16000g, 4°C. Separated serum was aliquoted and stored frozen at -80°C. Dosing of indicated cytokines/chemokines was performed using FlowCytomix Multiplex Kits (ebioscience) on an LSRII flow cytometer and according to the manufacturer protocol. Dosing of IFNα and IFNβ was done using VeriKine Mouse Interferon ELISA Kits (PBL Assay Science).

#### Human

Human cytokines were measured using a customized Milliplex Multiplex Assay Kit (EMD Millipore) for IFNα2, MCP-1, and IFNγ, and a customized ProcartaPlex Multiplex Immunoassay (eBioscience) for TNF, IL-6, CCL3, CCL4, and CXCL10. Assays were run on a Luminex MAGPIX Machine with Luminex xPONENT (version 4.2) software and analysis conducted with the MILLIPLEX Analyst software (version 3.5.5.0).

### Two-photon preparation, imaging and analysis

*In vivo* imaging of bone marrow was conducted as described previously [87]. Briefly, mice were anesthetized and the medial region of the tibial bone was surgically exposed, and carefully thinned to 100 micron thickness using a microdrill. Tissue was stabilized and mice were kept warm during imaging. Images were collected on an Olympus FV-1000MPE upright laser scanning microscope using a MaiTai DeepSee Laser (Spectraphysics). To visualize CD169 cells, mice were intravenously injected with 5μg of anti-CD169 (SER4) antibody conjugated to PE (BD Pharmingen), 16 hours prior to imaging [88]. All procedures were approved by the Institutional Animal Care and Use Committee.

#### Analysis

4D image sets were analyzed on Volocity 6.3 (Improvision). For cell-contact duration between pDCs and CD169^+^ cells, analysis was conducted by tallying time durations for all pDCs in the field in contact with CD169^+^ myeloid cells. Data was pooled from 3 or more mice from independent experiments. Supplementary movies were assembled using After Effects© (Adobe).

### Statistics

Statistical significance was calculated using an unpaired or a paired (human study) Student *t* test with GraphPad Prism software and two-tailed *P* values are given as: (*) *P*<0.1; (**) *P*<0.01; and (***) *P*<0.001; (ns) *P*>0.1. All *p* values of 0.05 or less were considered significant and are referred to as such in the text.

## Supporting Information

S1 Table Comparisons of variations of (**a**) cytokines and chemokines, (**b**) proportions of blood leukocyte subsets and (**c**) expression of activation/differentiation markers in human patients (enrollment/follow up) versus mice *(Py*-infected versus naive).
(PDF)

S2 Table Complete list of antibodies utilized in the study. (PDF)

S1 Fig **(A)** Table summarizes recorded clinical parameters in the severely infected patients enrolled in the study with schematic of study design. (**B**) WT B6 mice were inoculated i.v. with either 2x10^3^ (n = 10), 2x10^4^ (n = 29) or 2x10^5^ (n = 60) *Py 17X YM* iRBCs. (**A**) Survival and blood parasitemia of *Py*-infected mice over indicated times. (**C**) Kinetic of IFNβ levels in the blood in the first 48 hours post infection (n = 3). (**D**) 1.5 and/or 4.5 days post-infection (n = 5-9/condition), blood leucocytes were stained with mAbs against CD45, CD11b, Ly6C, NKp46, Ly6G, BST2, SiglecH or CD3, CD8, CD4, Foxp3, CD19 and the frequencies of each blood leukocyte (CD45^+^) subset determined. **(E)** Flow cytometry gating strategy applied to identify different cell subsets (Myeloid: monocytes and dendritic cells, Lymphoid:CD8, CD4 T and NK cells) found in patient PBMCs. (**F**) Frequency of viable cells in each individual patient PBMC sample. (**G**) Frequencies among live cells of indicated blood monocyte subsets (based on CD14 and CD16 expression), blood DCs subsets (mDCs and pDCs), NK cells, and T (CD4 and CD8) cells at enrollment versus follow-up. (**H—J**) Activation phenotype of blood DC subsets (mDCs and pDCs) (H), myeloid CD16^+^CD14^+^ inflammatory and CD16^+^CD14^dim^ patrolling monocyte subsets (I), and T (CD4 and CD8) cells (J) in individual patient PBMCs at enrollment versus follow up. Each open symbol represents an individual patient, enrollment (blue), follow-up (black). (**K**) Overlay of representative FACS histograms/dot plot for indicated activation markers expressed by Ly6C^+^ monocytes, NK cells, and pDCs in infected (day 1.5) versus uninfected WT mice (n = 3–14). P-values are indicated with * p<0.05, ** p<0.01.
(JPG)

S2 Fig **(A)** Representative FACS plot of chimerism in the spleen of 6 weeks reconstituted WT/*Ifnar1* ^*-/-*^ mixed bone marrow chimeras. (**B**) Representative FACS contour plots showing expression of indicated activation marker on blood Ly6C^+^ monocyte and NK cells 1.5 days after *Py* infection, in WT and *Ifnar1* ^*-/-*^ mice (upper panel, n = 6-10/genotype), or in WT and *Ifnar1* ^*-/-*^ cells mixed bone marrow chimeras (lower panel, n = 7).
(JPG)

S3 Fig **(A)** FACS gating strategy of pDCs in the different tissues analyzed is shown. Spleen, blood, and bone marrow cells were stained with the lineage markers CD11b, CD3, CD19, NK1.1, Ly6C, BST2 and Siglec-H. In spleen, an overlay of pDC (black) expression of B220/CD11c and of CD11b/Ly6C on whole cells (grey) is shown (n = 5). (**B**) Levels of IFNβ measured in the blood and bone marrow of day 1.5 *Py*-infected or uninfected WT mice (n = 3-14/condition). (**C**) Levels of IFNβ measured in the blood of DT-treated *Py*-infected WT, *Bdca2-Dtr* ^*+/-*^ or uninfected mice (n = 3-11/genotype). **(D)** Frequency of Ly6C^+^ monocytes and NK cells in the blood of DT-treated *Py*-infected *Bdca2-Dtr* ^*+/-*^ or WT B6 mice (n = 3-15/genotype). **(E)** DT-treated (every other day, starting 12 hours prior *Py* infection) *Bdca2-Dtr* ^*+/-*^ or WT B6 mice were inoculated with 2x10^5^ *Py 17X YM* iRBCs and survival was measured over time (n = 26-31/genotype). **(F)** Overlay of CXCR3, CCR2 and CCR5 expression in pDCs (black) compared to all CD45^+^ cells (grey) in the bone marrow of uninfected mice (n = 3/genotype). Experiments were replicated 2–4 times. P-values are indicated when applicable.
(JPG)

S4 Fig WT, *Myd88* ^*-/-*^ or *Sting* ^*Gt/Gt*^ mice were inoculated i.v. with 2x10^5^ *Py 17X YM* iRBCs. 1.5 days later, **(A)** levels of IFNβ in the bone marrow of WT or *Sting* ^*Gt/Gt*^ or uninfected control was measured (n = 3-10/genotype). **(B)** Frequency of YFP+ pDCs in bone marrow, blood, and spleen of *Py*-infected WT or *Sting* ^*Gt/Gt*^ *Ifnb-Yfp* ^*+/+*^ reporter mice (n = 3-8/genotype). (**C**) Blood cells were stained for the cell-surface lineage markers CD11b, Ly6C, NKp46, CD45, and frequencies of Ly6C^+^ monocytes and NK cells among CD45^+^ cells in the blood of *Py*-infected compared to uninfected (WT) mice is show (n = 3-8/genotype). Experiments were replicated 2–3 times. P-values are indicated when applicable.
(JPG)

S5 Fig Representative FACS plot of chimerism in the blood of indicated 6 weeks reconstituted mixed bone marrow chimeras. (JPG)

S6 Fig **(A)** DT-treated WT and *Cd169-Dtr* ^*+/-*^ *Ifnb-Yfp* ^*+/+*^ reporter mice (n = 3/condition) were inoculated i.v. with 2x10^5^ *Py 17X YM* iRBCs and bone marrow, blood, or spleen cells were stained with the lineage markers CD11b, BST2 and Siglec-H. Frequencies of pDCs among CD45^+^ cells is shown in uninfected and day 1.5 *Py*-infected mice. Bar graphs summarize the FACS data. **(B)** Frequencies of YFP^+^ pDCs in the bone marrow of *Py*-infected DT-treated or untreated *Cd169-Dtr* ^*+/-*^ *Ifnb-Yfp* ^*+/+*^ mice, and clodronate or control liposomes WT mice (n = 4-7/condition). **(C)** Activation profiles of Ly6C^+^ monocytes and NK cells using indicated markers in DT-treated WT or *LysM-Dtr* ^*+/-*^ mice (n = 3/genotype). Experiments were replicated 2–4 times. P-values are indicated when applicable.
(JPG)

S1 Movie Montage of time-lapse movies of pDCs (green), CD169^+^ cells (red) within the tibia bone marrow parenchyma in naïve *Ptcra-Gfp* ^*+/+*^ mice. (MOV)

S2 Movie Montage of time-lapse movies of pDCs (green), CD169^+^ cells (red) within the tibia bone marrow parenchyma in *Ptcra-Gfp* ^*+/+*^mice 36 hours following *Py* infection. (MOV)

S3 Movie Montage of time-lapse movies of pDCs (green), CD169^+^ cells (red) within the tibia bone marrow parenchyma in *Ifnb-Yfp* ^*+/+*^ mice 36 hours following *Py* infection. (MOV)
