# Supplementary material for: STING-Licensed Macrophages Prime Type I IFN Production by Plasmacytoid Dendritic Cells in the Bone Marrow during Severe Plasmodium yoelii Malaria
Source: PLoS Pathog. 2016 Oct 28;12(10):e1005975. doi: 10.1371/journal.ppat.1005975 (PMC5085251; doi:10.1371/journal.ppat.1005975)
Supplement: S1 Table — Comparisons of variations of (a) cytokines and chemokines, (b) proportions of blood leukocyte subsets and (c) expression of activation/differentiation markers in human patients (enrollment/follow up) versus mice (Py-infected versus naive). (PDF) [file ppat.1005975.s001.pdf]

A **Variations plasma cytokines and chemokines levels in human patients (admission/follow-up) and mice (naïve/infected)**

Plasma Cytokines & Chemokines	IFNα	IFNγ	TNFα	IL-6	CCL2	CCL3	CXCL1	CXCL10
Human	**ns**	**+**	**+**	**+**	**+**	**+**	**+**	**+**
Mouse (d1.5)	**+**	**+**	**+**	**ns**	**+**	**+**	**+**	**+**
Mouse (d4.5)	**ns**	**+**	**+**	**+**	**+**	**+**	**+**	**+**

B **Variations in blood frequencies of indicated cell subset among CD45⁺ leukocytes in human patients and mice**

**Monocytes**	Human	$CD14^+CD16^-$	$CD14^+CD16^+$	$CD14^{dim}/CD16^+$
		**+**	**+**	**ns**
	Mouse (d1.5&4.5)	$Ly6C^{hi}$		$Ly6C^{lo}$
		**+**		**nd**
**NK cells**	Human	$CD16^+56^+$		
		**ns**		
	Mouse (d1.5&4.5)	$NK1.1^+NKp46^+$		
		**+**		
**pDCs**	Human	$CD123^+CD11c^-HLA\text{-}DR^+CD14^-CD16^-$		
		**+**		
	Mouse (d1.5&4.5)	$BST2^+SiglecH^+CD11b^{low}B220^+$		
		**+**		
**T cells**	Human	$CD8^+$	$CD4^+$	$Foxp3^+$ Tregs
		**-**	**ns**	**nd**
	Mouse (d4.5)	**ns**	**ns**	**ns**

C **Variations in frequency of blood cell subset expressing indicated marker in human patients and mice**

**$CCR2^+$ monocytes**			CD40	CD86	MHC-II	CD11c	ICAM-1	BST2	F4/80	Sca-1	IL-15Rα
	Human	$CD14^+CD16^-$	**+**	**-**	**-**	**-**	**+**	**+**	**nd**	**nd**	**nd**
	Mouse, d1.5	$Ly6C^{hi}$	**nd**	**++**	**++**	**++**	**++**	**++**	**++**	**++**	**++**
	d4.5		**nd**	**+**	**+**	**+**	**+**	**+**	**nd**	**+**	**+**
**NK cells**			CD69	CD57	NKG2D	Ki67	Granzyme B	IFNγ			
	Human	$CD56^+CD16^+$	**+**	**ns**	**nd**	**+**	**nd**	**nd**			
	Mouse, d1.5	$NKp46^+NK1.1^+$	**++**	**nd**	**++**	**ns**	**++**	**++**			
	d4.5		**+**	**nd**	**+**	**ns**	**++**	**+**			
**Dendritic cells**	**pDCs**			CD86	HLA-DR	BST2	**mDC**		CD86	HLA-DR	BST2
	Human	$CD123^+CD11c^-HLA\text{-}DR^+$		**+**	**+**	**+**	$CD11c^+CD123^-HLA\text{-}DR^+$		**-**	**-**	**+**
	Mouse, d1.5	$BST2^+SiglecH^+CD11b^{low}$		**+**	**nd**	**+**	**nd**				
	d4.5			**-**	**nd**	**-**	**nd**				
**T cells**	**$CD8^+$ T cells**	$CD62L^{low}$	CD69	CXCR3	KLRG1	Ki67	Tbet	IFNγ			
	Human	**+**	**+**	**nd**	**nd**	**+**	**ns**	**nd**			
	Mouse, d4.5	**ns**	**ns**	**+**	**+**	**+**	**+**				
	**$CD4^+$ T cells**	$CD62L^{low}$	CD69	CXCR3	CD44	Ki67	Tbet	IFNγ	CD127		
	Human	**+**	**ns**	**ns**	**nd**	**+**	**+**	**nd**	**nd**		
	Mouse, d4.5	**ns**	**ns**	**+**	**+**	**+**	**+**	**+**	**-**		
	**$Foxp3^+CD4^+$ Treg cells**		ICOS	Ki67							
	Human	nd									
	Mouse, d4.5		**+**	**+**							

Table S1
