# Supplementary material for: STING-Licensed Macrophages Prime Type I IFN Production by Plasmacytoid Dendritic Cells in the Bone Marrow during Severe Plasmodium yoelii Malaria
Source: PLoS Pathog. 2016 Oct 28;12(10):e1005975. doi: 10.1371/journal.ppat.1005975 (PMC5085251; doi:10.1371/journal.ppat.1005975)
Supplement: S2 Table — (PDF) [file ppat.1005975.s002.pdf]

Antibody Target	Fluorochrome	Clone	Purchased from
**FACS (mouse)**			
B220	PE-Cy7, PE	RA3-6B2	eBioscience
BST2 (CD317)	APC	eBio927	eBioscience
CCR5	Biotin	C34-3448	BD Bioscience
CCR7	Biotin	4B12	eBioscience
CD11b	eFluor450	M1/70	eBioscience
CD11c	PE, PE-Cy7	N418	eBioscience
CD127	APC, PerCP-Cy5.5	A7R34	eBioscience
CD19	Biotin, V510	1D3	BD Bioscience
CD25	APC, PE-Cy7	PC-61	BD Bioscience
CD3	Biotin, V510	145-2C11	BD Bioscience
CD4	PerCP-Cy5.5, APC, PE-Cy7	RM4-5	BD Bioscience
CD44	AF700, APC	IM7	BD Bioscience
CD45	AF700	30F11	eBioscience
CD45.1	FITC, Pacific Blue	A20	eBioscience
CD45.1	PE-CF594	A20	BD Bioscience
CD45.2	PE, PerCP-Cy5.5, AF700	104	BD Bioscience
CD62L	APC, PerCP-Cy5.5, AF700	MEL-14	BD Bioscience
CD69	PerCP-Cy5.5	H1.2F3	eBioscience
CD8	PE-Cy7, PE, AF700, V500	53-6.7	BD Bioscience
CD86	AF700	GL1	BD Bioscience
CXCR3	PerCP-Cy5.5, PE	CXCR3-173	eBioscience
F480	APC, Biotin	BM8	eBioscience
Foxp3	eFluor450	FJK165	eBioscience
Granzyme B	APC	MHGB05	Invitrogen
ICAM-1 (CD54)	Biotin	YN1.1.7.4	eBioscience
ICOS (CD278)	Biotin	7E.17G9	eBioscience
IFNγ	PE, PE-Cy7	XMG1.2	BD Bioscience
IL-15Rα	PE	DNT15Rα	eBioscience
Ki67	FITC	SolA15	eBioscience
KLRG1	FITC, PE-Cy7	2F1	eBioscience
Ly6C	FITC, Alexa 700	AL-21	eBioscience
Ly6G	PE, PerCP-Cy5.5, AF700	1A8	BD Bioscience
MHCII	AF700, Biotin	M5/114.15.2	eBioscience
NK1.1	PE, PE-Cy7, Biotin	PK136	eBioscience
NKG2D	PE-Cy7	CX5	eBioscience
NKp46	Pacific Blue, PE	29A1.4	BD Bioscience
Sca-1	AF700	D7	eBioscience
Siglec-H	PE-Cy7	eBio44c	eBioscience
Tbet	PE-Cy7	4B10	eBioscience
**FACS (human)**			
CD11c	PE-Cy7	B-ly6	BD Biosciences
CD123	PerCP-Cy5.5	7G3	BD Biosciences
CD14	Alexa Fluor 700	M5E2	BD Biosciences
CD16	APC-Cy7	3G8	BD Biosciences
CD19	Biotin	HIB19	BD Biosciences
CD3	Biotin	UCHT1	BD Biosciences
CD317 (BST2)	PE	2688	eBioscience
CD4	Alexa Fluor 700	RPAT4	BD Biosciences
CD40	BUV395	5C3	BD Biosciences
CD45RO	FITC	UCHL1	BD Biosciences
CD54 (ICAM-1)	PE-Cy5	HA58	BD Biosciences
CD56	Biotin	B159	BD Biosciences
CD57	PE	NK-1	BD Biosciences
CD62L	APC	DREG-56	BD Biosciences
CD69	BUV395	FN50	BD Biosciences
CD8	PE-CF594	RPA-T8	BD Biosciences
CD86	PE-CF594	FUN-1	BD Biosciences
CX3CR1	APC	2A9-1	eBioscience
HLA-DR	FITC	TU36	BD Biosciences
Ki67	V450	B56	BD Biosciences
Perforin	PerCP	B-D48	Diaclone
Tbet	PE-Cy7	4B10	eBioscience
**Secondary**			
Streptavidin	PE-Cy5		BD Biosciences
Streptavidin	V450		BD Biosciences
Streptavidin	eFluor 710		eBioscience
Streptavidin	eFluor 450		eBioscience
Streptavidin	PE-Cy7		BD Biosciences
Streptavidin	APC		BD Biosciences
Streptavidin	PerCP-Cy5.5		eBioscience
Streptavidin	V500		BD Biosciences
anti-Histidine tag	Biotin	6G2A9	GenScript
**Intravital Microscopy**			
CD169	PE	SER-4	eBioscience

Table S2
