# Supplementary figures and images for: STING-Licensed Macrophages Prime Type I IFN Production by Plasmacytoid Dendritic Cells in the Bone Marrow during Severe Plasmodium yoelii Malaria
Source: PLoS Pathog. 2016 Oct 28;12(10):e1005975. doi: 10.1371/journal.ppat.1005975 (PMC5085251; doi:10.1371/journal.ppat.1005975)

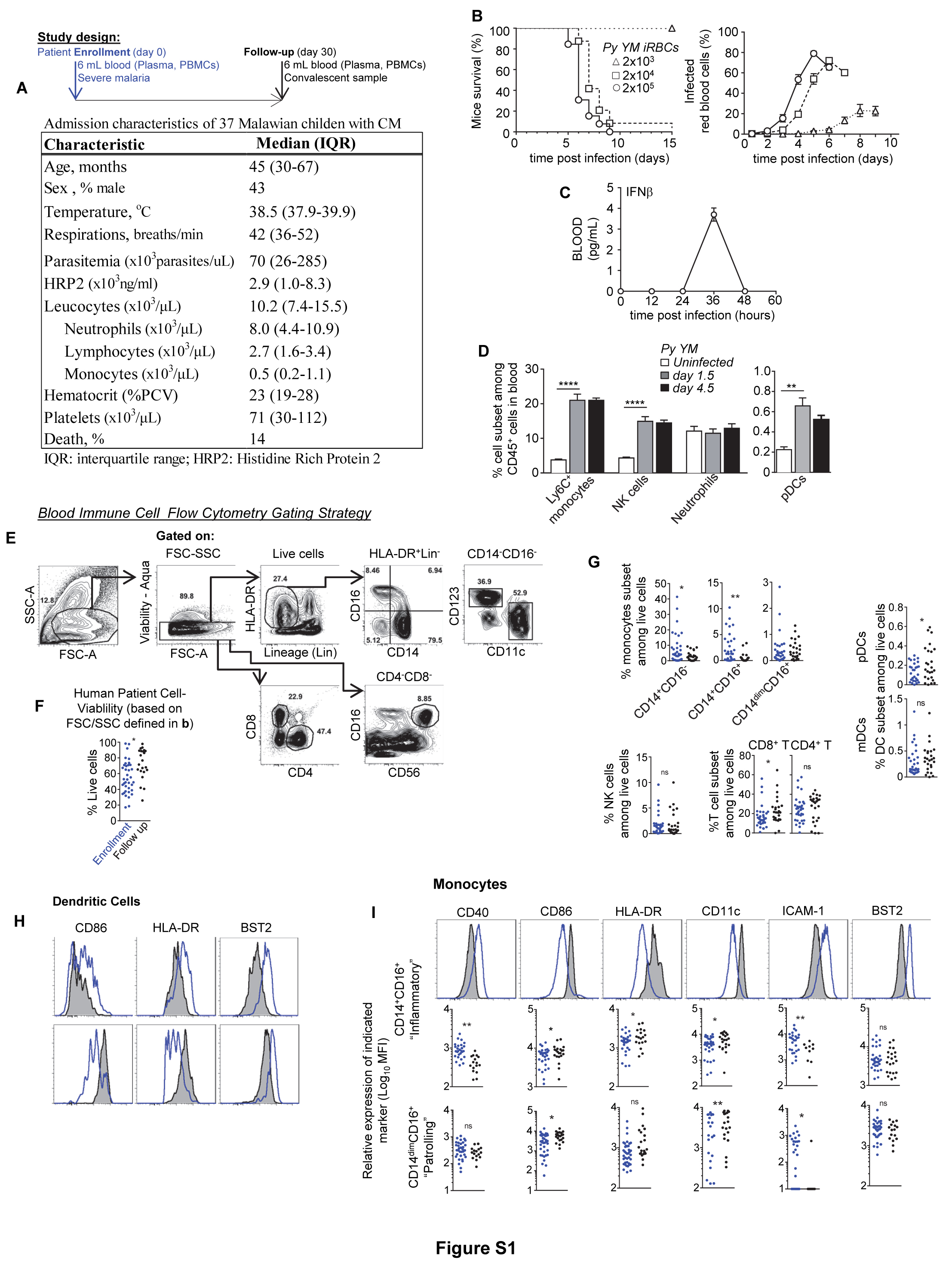

Supplement: S1 Fig — (A) Table summarizes recorded clinical parameters in the severely infected patients enrolled in the study with schematic of study design. (B) WT B6 mice were inoculated i.v. with either 2x10^3^ (n = 10), 2x10^4^ (n = 29) or 2x10^5^ (n = 60) Py 17X YM iRBCs. (A) Survival and blood parasitemia of Py-infected mice over indicated times. (C) Kinetic of IFNβ levels in the blood in the first 48 hours post infection (n = 3). (D) 1.5 and/or 4.5 days post-infection (n = 5-9/condition), blood leucocytes were stained with mAbs against CD45, CD11b, Ly6C, NKp46, Ly6G, BST2, SiglecH or CD3, CD8, CD4, Foxp3, CD19 and the frequencies of each blood leukocyte (CD45+) subset determined. (E) Flow cytometry gating strategy applied to identify different cell subsets (Myeloid: monocytes and dendritic cells, Lymphoid:CD8, CD4 T and NK cells) found in patient PBMCs. (F) Frequency of viable cells in each individual patient PBMC sample. (G) Frequencies among live cells of indicated blood monocyte subsets (based on CD14 and CD16 expression), blood DCs subsets (mDCs and pDCs), NK cells, and T (CD4 and CD8) cells at enrollment versus follow-up. (H—J) Activation phenotype of blood DC subsets (mDCs and pDCs) (H), myeloid CD16+CD14+ inflammatory and CD16+CD14dim patrolling monocyte subsets (I), and T (CD4 and CD8) cells (J) in individual patient PBMCs at enrollment versus follow up. Each open symbol represents an individual patient, enrollment (blue), follow-up (black). (K) Overlay of representative FACS histograms/dot plot for indicated activation markers expressed by Ly6C+ monocytes, NK cells, and pDCs in infected (day 1.5) versus uninfected WT mice (n = 3–14). P-values are indicated with * p<0.05, ** p<0.01. (JPG) [file ppat.1005975.s003.jpg]

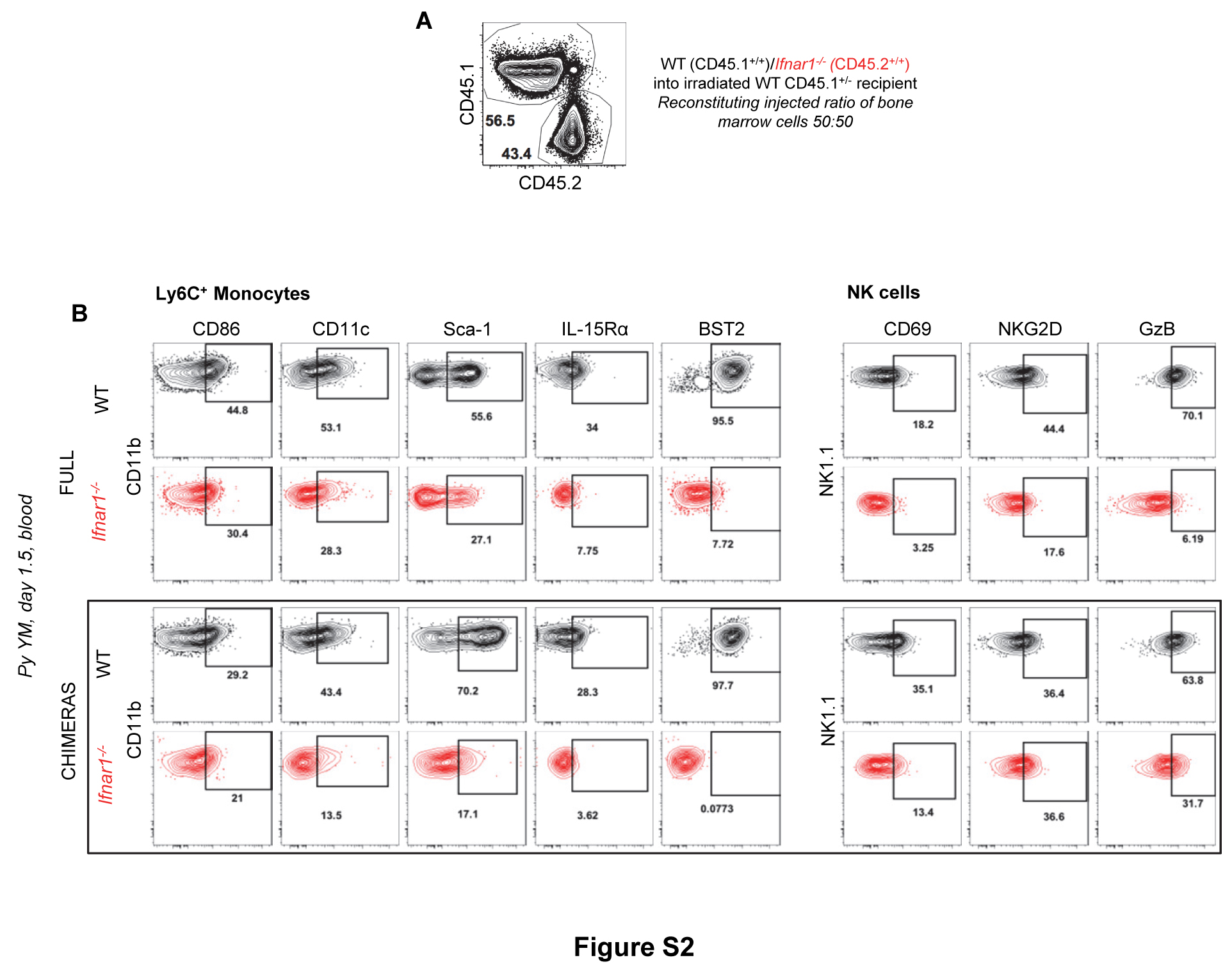

Supplement: S2 Fig — (A) Representative FACS plot of chimerism in the spleen of 6 weeks reconstituted WT/Ifnar1 -/- mixed bone marrow chimeras. (B) Representative FACS contour plots showing expression of indicated activation marker on blood Ly6C+ monocyte and NK cells 1.5 days after Py infection, in WT and Ifnar1 -/- mice (upper panel, n = 6-10/genotype), or in WT and Ifnar1 -/- cells mixed bone marrow chimeras (lower panel, n = 7). (JPG) [file ppat.1005975.s004.jpg]

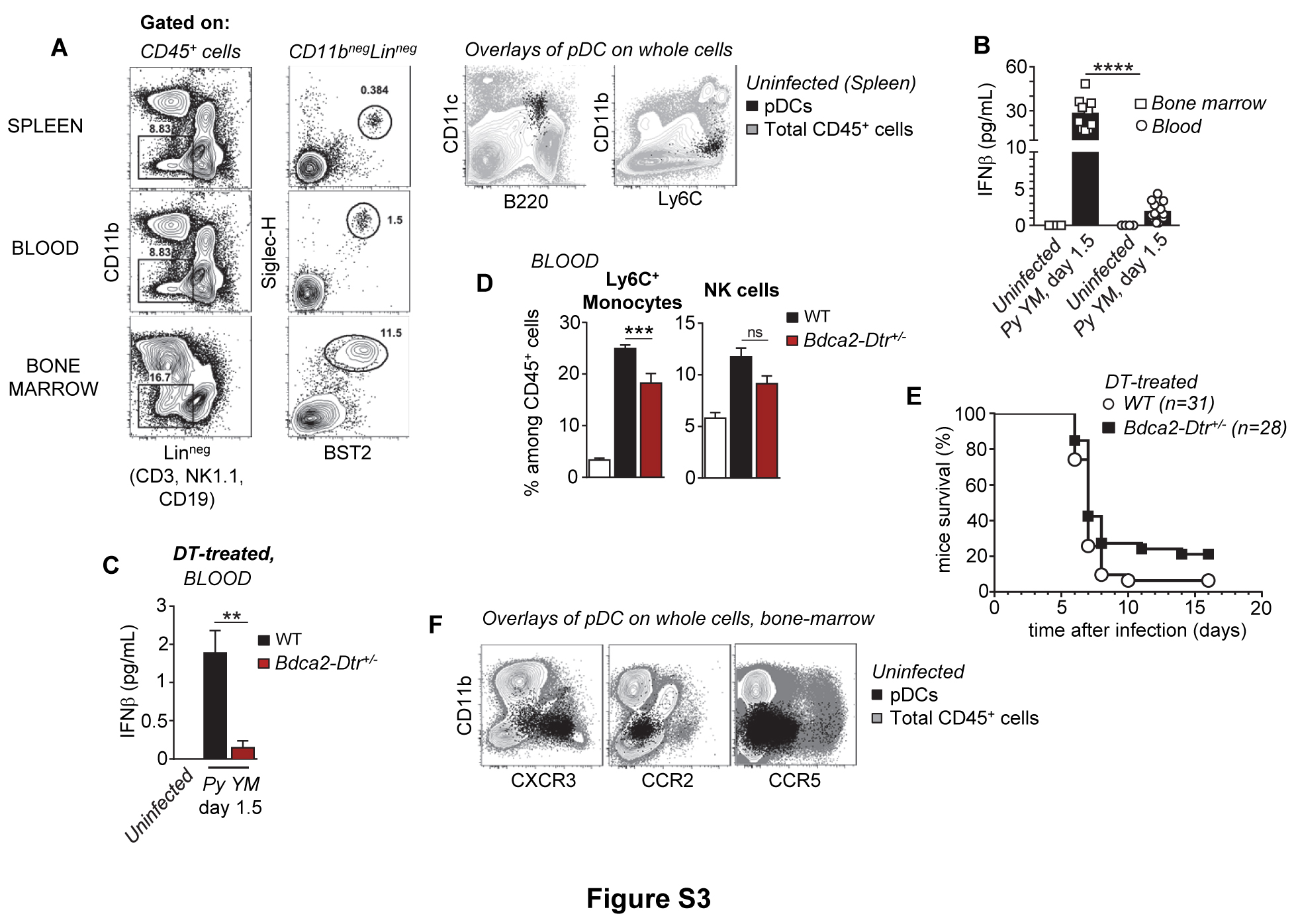

Supplement: S3 Fig — (A) FACS gating strategy of pDCs in the different tissues analyzed is shown. Spleen, blood, and bone marrow cells were stained with the lineage markers CD11b, CD3, CD19, NK1.1, Ly6C, BST2 and Siglec-H. In spleen, an overlay of pDC (black) expression of B220/CD11c and of CD11b/Ly6C on whole cells (grey) is shown (n = 5). (B) Levels of IFNβ measured in the blood and bone marrow of day 1.5 Py-infected or uninfected WT mice (n = 3-14/condition). (C) Levels of IFNβ measured in the blood of DT-treated Py-infected WT, Bdca2-Dtr +/- or uninfected mice (n = 3-11/genotype). (D) Frequency of Ly6C+ monocytes and NK cells in the blood of DT-treated Py-infected Bdca2-Dtr +/- or WT B6 mice (n = 3-15/genotype). (E) DT-treated (every other day, starting 12 hours prior Py infection) Bdca2-Dtr +/- or WT B6 mice were inoculated with 2x10^5^ Py 17X YM iRBCs and survival was measured over time (n = 26-31/genotype). (F) Overlay of CXCR3, CCR2 and CCR5 expression in pDCs (black) compared to all CD45+ cells (grey) in the bone marrow of uninfected mice (n = 3/genotype). Experiments were replicated 2–4 times. P-values are indicated when applicable. (JPG) [file ppat.1005975.s005.jpg]

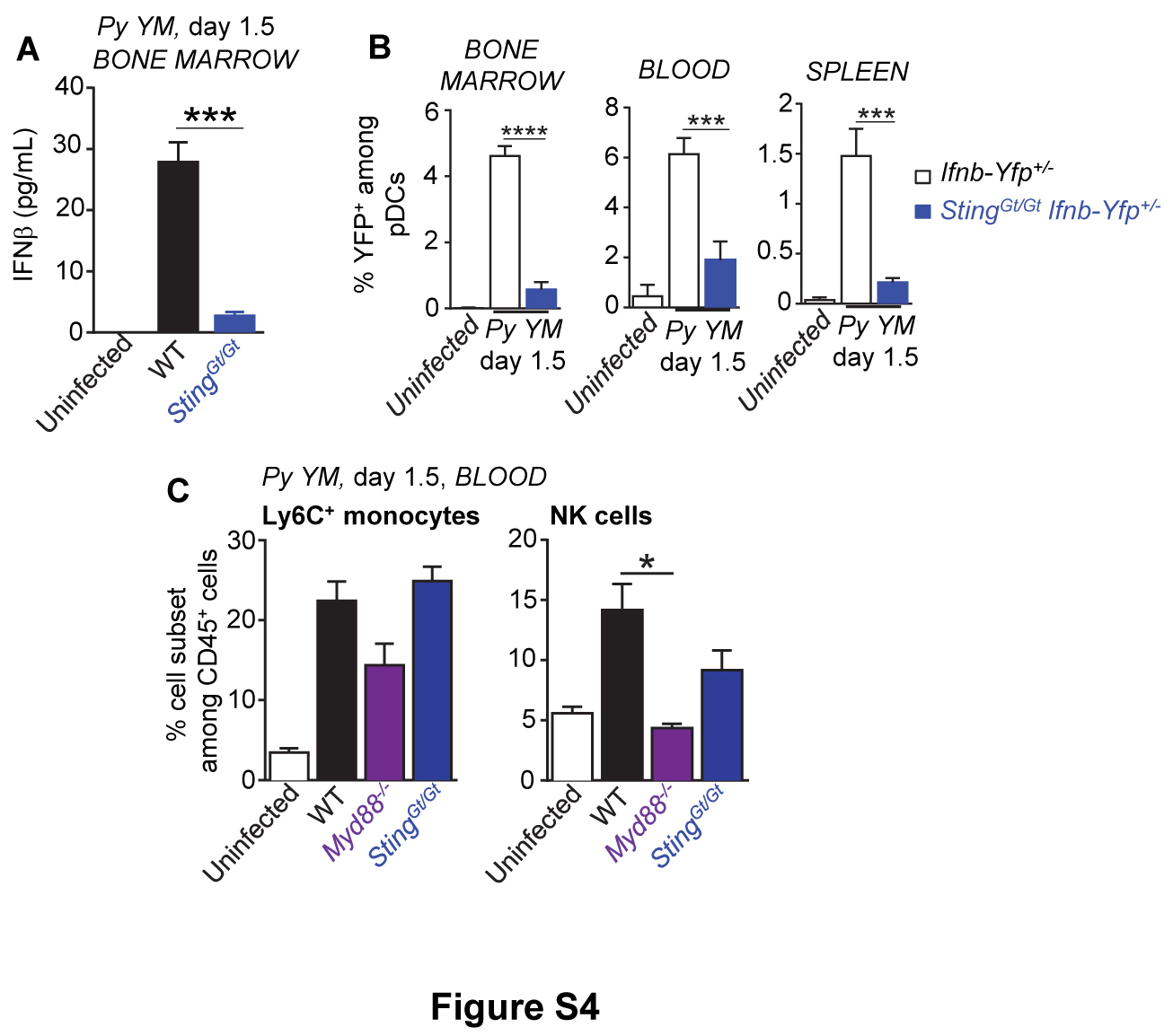

Supplement: S4 Fig — 1.5 days later, (A) levels of IFNβ in the bone marrow of WT or Sting Gt/Gt or uninfected control was measured (n = 3-10/genotype). (B) Frequency of YFP+ pDCs in bone marrow, blood, and spleen of Py-infected WT or Sting Gt/Gt Ifnb-Yfp +/+ reporter mice (n = 3-8/genotype). (C) Blood cells were stained for the cell-surface lineage markers CD11b, Ly6C, NKp46, CD45, and frequencies of Ly6C+ monocytes and NK cells among CD45+ cells in the blood of Py-infected compared to uninfected (WT) mice is show (n = 3-8/genotype). Experiments were replicated 2–3 times. P-values are indicated when applicable. (JPG) [file ppat.1005975.s006.jpg]

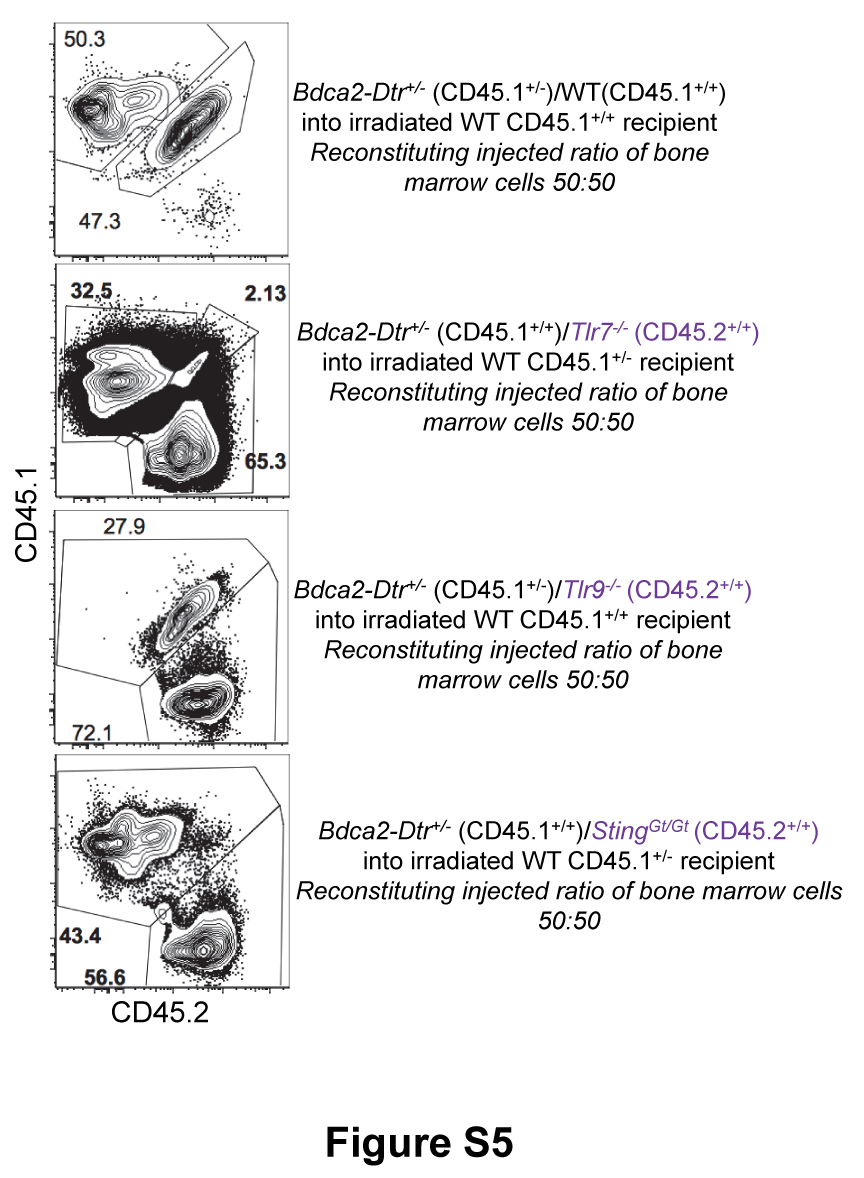

Supplement: S5 Fig — (JPG) [file ppat.1005975.s007.jpg]

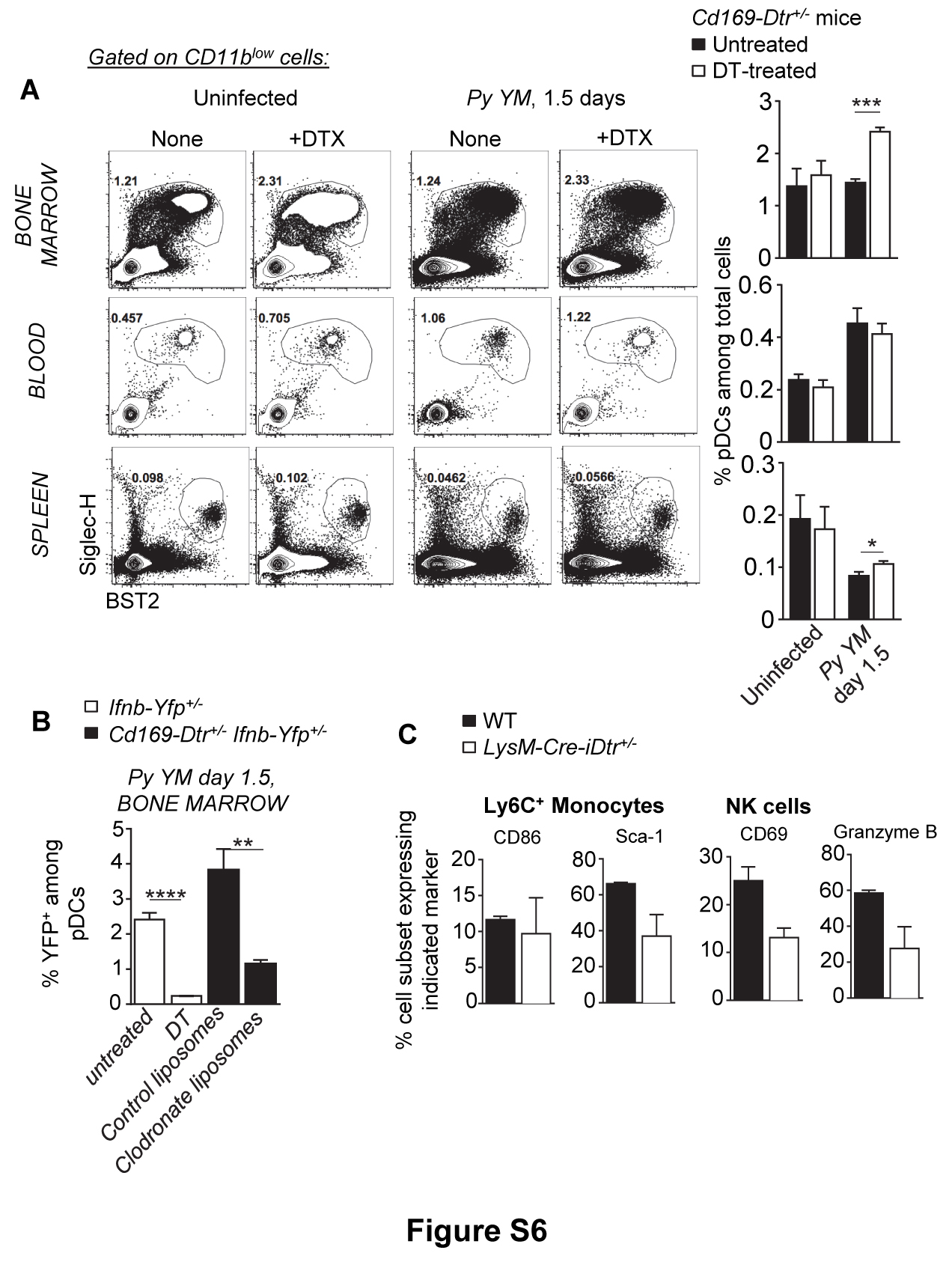

Supplement: S6 Fig — (A) DT-treated WT and Cd169-Dtr +/- Ifnb-Yfp +/+ reporter mice (n = 3/condition) were inoculated i.v. with 2x10^5^ Py 17X YM iRBCs and bone marrow, blood, or spleen cells were stained with the lineage markers CD11b, BST2 and Siglec-H. Frequencies of pDCs among CD45+ cells is shown in uninfected and day 1.5 Py-infected mice. Bar graphs summarize the FACS data. (B) Frequencies of YFP+ pDCs in the bone marrow of Py-infected DT-treated or untreated Cd169-Dtr +/- Ifnb-Yfp +/+ mice, and clodronate or control liposomes WT mice (n = 4-7/condition). (C) Activation profiles of Ly6C+ monocytes and NK cells using indicated markers in DT-treated WT or LysM-Dtr +/- mice (n = 3/genotype). Experiments were replicated 2–4 times. P-values are indicated when applicable. (JPG) [file ppat.1005975.s008.jpg]
